# A Novel Strategy for Highly Efficient Heterologous Expression of Carbonic Anhydrase in *Yarrowia lipolytica*

**DOI:** 10.3390/ijms27104224

**Published:** 2026-05-09

**Authors:** Guowei Zhao, Mengqin Zhu, Huanhuan Li, Liangcheng Jiao, Yunchong Li, Kaixin Yang, Wenping Wei, Min Yang, Yunjun Yan

**Affiliations:** Key Laboratory of Molecular Biophysics of the Ministry of Education, College of Life Science and Technology, Huazhong University of Science and Technology, Wuhan 430074, China; zhaoguowei@hust.edu.cn (G.Z.); jiaoliangcheng@gmail.com (L.J.); liyunchong77@hust.edu.cn (Y.L.); ykxllq@163.com (K.Y.); wenping1204@126.com (W.W.); ymyangmin@hust.edu.cn (M.Y.)

**Keywords:** *Yarrowia lipolytica*, markerless recombination, carbonic anhydrase, heterologous expression, CO_2_ mineralization

## Abstract

Carbonic anhydrases (CAs) efficiently catalyze CO_2_ reversible hydration, critical for carbon capture and sequestration (CCS), but naturally low yield limits industrial use. *Yarrowia lipolytica*, an unconventional yeast, is an ideal heterologous expression host with robust adaptability, post-translational modification capacity, and versatile genetic tools. In this study, 10 α-, β-, and γ-class CAs were successfully expressed in *Y. lipolytica*, and two top-performing candidates were identified: *Methanosarcina mazei* γ-CA (MmaCA) and *Sulfurihydrogenibium azorense* α-CA (SazCA). Their production was further optimized via promoter and gene dosage adjustment, cultural condition optimization and auxiliary protein co-expression. The optimized intracellular MmaCA activity reached 960 U/mL (64.42-fold improvement), and the extracellular SazCA activity peaked at 925 U/mL (70.08-fold enhancement). CO_2_ mineralization experiments confirmed both recombinant CAs significantly accelerated CaCO_3_ precipitation, demonstrating a promising CCS application potential. To our knowledge, this is the first systematic investigation of CA heterologously expressed in *Y. lipolytica*, providing a novel strategy for the highly efficient production of CAs to enable their application in industry.

## 1. Introduction

The continuous increase in atmospheric CO_2_ concentration caused by human industrial activities has become a major global environmental problem, leading to serious climate change such as global warming and sea level rise [1]. Carbon capture and sequestration (CCS) technology is considered an effective strategy to mitigate global warming, and the development of efficient and environmentally friendly CO_2_ conversion technologies has attracted extensive attention from researchers worldwide [2].

Carbonic anhydrase (CA, EC 4.2.1.1), a ubiquitous metalloenzyme found across the three biological domains of archaea, eubacteria, and eukaryotes, predominantly utilizes Zn^2+^ as its active center to efficiently catalyze the reversible hydration reaction of CO_2_ [3]. This enzymatic process can be described by the following reaction equation:

CO_2_ + H_2_O ⇋ HCO_3_^−^ + H^+^, (1)

Under spontaneous conditions, the reaction proceeds at a rate of approximately 0.15 s^−1^, which is insufficient to meet the rapid conversion demands between CO_2_ and HCO_3_^−^ required for cellular metabolic activities. Consequently, the normal cellular metabolism is hindered, impairing the ability of cells to respond promptly and appropriately to environmental changes [4,5]. In contrast, CA, which catalyzes this reaction, elevates the reaction rate to a remarkable 10^4^ to 10^6^ s^−1^ [6], positioning it among the fastest known biological catalysts in nature. Therefore, CA is indispensable for the growth and metabolism of the vast majority of organisms, participating in a wide array of vital physiological processes such as respiration, photosynthesis, pH regulation, and maintenance of CO_2_ homeostasis, electrolyte secretion in various tissues and organs, bone resorption and calcification, and biomineralization [5,7]. In mammals, CAs play important roles in cellular respiration, acid-base regulation, electrolyte secretion, bone resorption and calcification, as well as biosynthetic reactions that require HCO_3_^−^ as a substrate (e.g., fat synthesis, gluconeogenesis, and urea production) [8,9,10]. In higher plants and microalgae, CA primarily contributes to photosynthesis and the CO_2_-concentrating mechanism (CCM), which enhances the carbon fixation efficiency of ribulose-1,5-bisphosphate carboxylase/oxygenase (Rubisco) by accumulating high concentrations of CO_2_ within cells. This mechanism bolsters CO_2_ fixation and enables organisms to survive under conditions where CO_2_ concentrations limit photosynthesis [11,12,13]. Furthermore, in protozoa, fungi, and bacteria, the catalytic action of CA is crucial for maintaining intracellular pH homeostasis.

These carbonic anhydrases are widely distributed in nature, and there are numerous types. To date, known carbonic anhydrases can be classified into eight distinct families: α, β, γ, δ, ζ, ƞ, θ, and ι. These families are phylogenetically unrelated and exhibit minimal sequence or structural similarity [14], which serves as a typical example of convergent evolution. Currently, research on CAs has primarily focused on three types: α, β, and γ. α-CA is the earliest discovered and most abundant CA family, commonly found in animals, higher plants, algae, protozoa, fungi, and bacteria [15]. β-CA was first identified in the chloroplasts of higher plants in 1990 and has since been found in algae, fungi, bacteria, and archaea [16,17]. γ-CA was first discovered in 1994 in *Methanosarcina thermophila* and later identified as being widely present in bacteria and the mitochondria of plants [18,19,20].

Owing to their unique structural diversity and exceptional catalytic properties, CAs have attracted extensive research interest across multiple disciplines. The biochemical properties of CAs vary significantly depending on their organismal source. Notably, CAs derived from extremophilic microorganisms, such as thermophiles and alkaliphiles, frequently demonstrate superior thermostability [21,22], broader pH tolerance [23], and in many cases, exceptional catalytic efficiency [22,24] compared to their mammalian counterparts. These attributes make them particularly promising candidates for industrial applications, such as carbon capture and sequestration under harsh process conditions [22,25].

Renowned for their high catalytic efficiency, relative stability, and biodegradability, CA has emerged as a research focus in various application domains, including drug design, biosensors, and CO_2_ capture [1,26,27]. To date, 15 distinct CA isoenzymes have been identified in humans, each exhibiting unique structural characteristics, properties, and tissue distributions, yet all play pivotal roles in physiological functions. Consequently, they have been extensively studied for the treatment of various diseases, such as cancer, obesity, epilepsy, and glaucoma [28,29,30]. CO_2_ sensing, along with HCO_3_^−^ and pH sensing, is a critical component of numerous biological processes. Studies have shown that CA functions as a sensor in various physiological systems [31,32]. Recently, CCS technologies based on CA have garnered significant attention. By harnessing CA for CO_2_ capture and promoting biomimetic mineralization of CaCO_3_, these technologies aim to reduce CO_2_ emissions into the atmosphere, thereby mitigating global warming effects [2,5,27].

Although CAs have significant application value for the fixation of greenhouse gas CO_2_, most of the CAs currently studied are derived from mammals (such as human CAII and bovine erythrocyte CA), which are costly to obtain. In contrast, microbial sources, which could offer lower production costs, are relatively scarce. As a result, there has been an increasing number of reports on the microbial production of CAs. In recent years, many CA-producing microorganisms have been discovered with the advancement of genomic technologies; however, their growth conditions are often limited, and the yield of CAs is relatively low, making large-scale preparation challenging. For example, Chen et al. reported that the CA activity of microorganisms isolated directly from environmental samples ranges only from 0.09 to 1.26 U/mL [33]. Heterologous protein expression, as a well-established biotechnological approach, can effectively address these issues. Currently, various CAs have been successfully expressed heterologously, especially in *Escherichia coli* [34,35,36,37,38,39,40]. In addition, Human CAII was successfully expressed in *Saccharomyces cerevisiae* [41,42] and *Pichia pastoris* [43], respectively.

Research on the heterologous expression of CAs in prokaryotic expression systems is relatively abundant, while studies in eukaryotic expression systems are less common. Among eukaryotic systems, yeast presents distinct advantages, including the ability to perform appropriate post-translational modifications, rapid growth, straightforward genetic manipulation, scalable fermentation, high biomass concentration, and safe pathogen-free production [44].

*Yarrowia lipolytica* is an unconventional yeast that significantly differs from traditional model organisms, such as *S. cerevisiae*, in terms of evolution, physiology, and metabolism. It initially attracted attention due to its remarkable lipid degradation and protein hydrolysis capabilities. *Y. lipolytica* is a strictly aerobic oil-producing yeast that thrives at temperatures between 25 °C and 30 °C and exhibits strong tolerance to varying salt concentrations and pH levels [45]. Furthermore, it is rich in various intracellular and extracellular enzyme systems, including lipases, proteases, and esterases, which endow it with significant substrate metabolism capabilities. This yeast can utilize hydrophilic substrates such as glucose, organic acids, and polyols, as well as metabolize a variety of hydrophobic substrates, including alkanes, fatty acids, and triglycerides [46,47]. *Y. lipolytica* is widely distributed in nature and in human environments, commonly found in meat products, dairy, seawater, salt marshes, wastewater, and oil-contaminated sites [48,49,50,51].

*Y. lipolytica*, a generally recognized as safe (GRAS) yeast, possesses distinct advantages as a heterologous protein expression host, including a broad carbon source spectrum that enables utilization of low-cost hydrophilic and hydrophobic substrates for growth and fermentation, a sophisticated post-translational modification system facilitating correct folding and modification of recombinant proteins analogous to higher eukaryotes, robust protein secretion capacity that simplifies the separation and purification of target proteins, and a well-characterized genome complemented by diverse developed genetic manipulation tools and expression vectors [45].

Utilizing* Y. lipolytica* for the expression of recombinant proteins offers significant advantages and has become an important chassis strain for production in the food and pharmaceutical industries. However, there have been few reports on the heterologous expression of CA in *Y. lipolytica*. In 2021, You et al. [52] introduced CA into *Y. lipolytica* to achieve the conversion of CO_2_ to HCO_3_^−^, while also overexpressing biotin carboxylase (BC) to channel HCO_3_^−^ into the fatty acid metabolic pathway, aiming to obtain higher fatty acid yields. However, a systematic investigation into the expression of CA had not yet been conducted. This work marks the first comprehensive effort to express CA from various sources and types in *Y. lipolytica*. Multiple strategies were employed to increase CA expression levels, and the potential of CA in CO_2_ mineralization was thoroughly validated.

## 2. Results

Based on the gene resources in our laboratory and the NCBI database, carbonic anhydrase (CA) genes from 10 different sources (predominantly sourced from microorganisms) were selected (Table 1), representing three types: α, β, and γ. The expression levels of these various CA genes in *Y. lipolytica* were investigated using the previously reported markerless integrative vector pUAxp7166 [53].

In this study, we employed a systematic multi-step optimization strategy to develop an efficient heterologous expression system for carbonic anhydrases in *Y. lipolytica*. First, we expressed and screened 10 CAs from α-, β-, and γ-classes to identify candidates with high expression potential. For the two top-performing enzymes (intracellular MmaCA and extracellular SazCA), we sequentially optimized promoter strength, gene dosage, and fermentation conditions to enhance their production. We further improved enzyme yields by co-expressing auxiliary proteins involved in protein folding and synthesis. Finally, we characterized the recombinant enzymes and evaluated their application potential in CO_2_ mineralization.

### 2.1. Preliminary Expression of CA in Y. lipolytica

**Figure 1:**
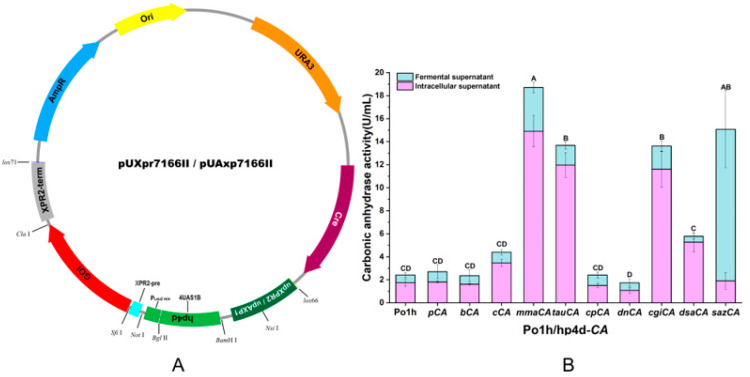
(**A**) Plasmid structure employed in this study. (**B**) Determination of the preliminary expressed enzyme activity of CA, with significance analysis based on total enzyme activity labeling.

To facilitate vector construction, the restriction sites of pUAxp7166 were optimized by direct replacement of the coding sequence (CDS) during plasmid assembly, generating the new template plasmid pUAxp7166II. This strategy allows flexible selection for the XPR2 signal peptide, which can be achieved by choosing either *Not* I or *Sfi* I restriction sites, as shown in Figure 1A.

The *pCA* fragment was obtained by PCR amplification of the Po1h genome. Except for *pCA*, all gene sequences listed in Table 1 were codon-optimized and synthesized into the pUC57 vector. The final PCR products were designed to carry *Sfi* I and *Cla* I restriction sites at their 5′ and 3′ ends, respectively, which were consistent with those in the integration vector. Following seamless cloning, or double digestion with these restriction enzymes, the CDS was integrated into the integration vector pUAxp7166II.

The plasmid pUAxp7166II (with CA gene) was linearized with the *Nsi* I restriction enzyme and introduced into the Po1h strain via the LiAc transformation method. Through Single Crossover Homologous Recombination (SCOHR), 10 CA genes were integrated into the upstream region of the *Axp1* gene, respectively. Subsequent induction with MO+Ura medium facilitated the excision of selection marker-containing irrelevant sequences, ultimately retaining only a *lox72* scar sequence.

Ten strains of Po1h/hp4d-CA were individually inoculated into 50 mL of BMSY medium, with Po1h serving as the control group. After 5 days of shake-flask cultivation, the CA enzyme activity was measured. The supernatant of the fermented broth, obtained by centrifugation, was used to determine the extracellular enzyme activity. The collected cells were subjected to cell disruption, and the supernatant from the subsequent centrifugation was analysed to assess the intracellular CA enzyme activity. The results are presented in Figure 1B.

The total intracellular and extracellular CA activities of the recombinant strains are summarized in Figure 1B. The enzymatic assay results revealed substantial variations in both intracellular and extracellular CA activities among the engineered yeast strains. In the extracellular CA activity assay, the supernatant of the recombinant strain Po1h/hp4d-*sazCA*, harboring the *sazCA* gene, exhibited significantly higher CA activity (13.2 ± 3.4 U/mL) compared to other strains. Additionally, recombinant strains expressing *mmaCA*, *tauCA*, and *cgiCA* genes demonstrated elevated CA activities relative to the control strain Po1h, whereas other engineered strains displayed only trace levels of extracellular CA activity. In the intracellular CA activity assay, the recombinant strain Po1h/hp4d-*mmaCA* exhibited the highest intracellular CA activity (14.9 ± 1.37 U/mL), substantially exceeding its extracellular activity (3.8 ± 0.45 U/mL). Furthermore, strains expressing* tauCA*, *cgiCA*, and *dsaCA *also displayed relatively high intracellular CA activities, albeit lower than Po1h/hp4d-*mmaCA*. No significant differences were observed in intracellular CA activities among strains expressing *pCA*, *bCA*, *cCA*, *cpCA*, and *sazCA* genes. Notably, the strain expressing the *dnCA* gene exhibited lower intracellular CA activity than the control strain Po1h (1.75 ± 0.31 U/mL). On the other hand, MmaCA (γ-CA) from *Methanosarcina mazei* and SazCA (α-CA) from *Sulfurihydrogenibium azorense* demonstrated the highest total enzymatic activities, while other CAs exhibited relatively lower expression levels in *Y. lipolytica*. Notably, SazCA displayed the highest extracellular CA activity, whereas MmaCA exhibited the highest intracellular activity, suggesting efficient secretion of SazCA into the extracellular environment and predominant intracellular retention of MmaCA. These findings indicate significant differences in the expression levels of CAs from diverse origins or types in *Y. lipolytica*.

Based on the enzymatic activity assay results, we have selected γ-type CA (MmaCA) and α-type CA (SazCA) for further investigation, as these two enzymes exhibited the highest intracellular and extracellular activities, respectively, among the tested carbonic anhydrases.

### 2.2. High-Efficiency Heterologous Expression of MmaCA in Y. lipolytica

#### 2.2.1. Strategies for Promoting the Secretion of MmaCA

In previous experiments, MmaCA demonstrated notably high intracellular enzymatic activity, while a certain level of extracellular activity was also detected. To facilitate protein collection, several strategies were thus employed to enhance MmaCA secretion.

- Truncate the hydrophobic α-helix sequence

**Figure 2:**
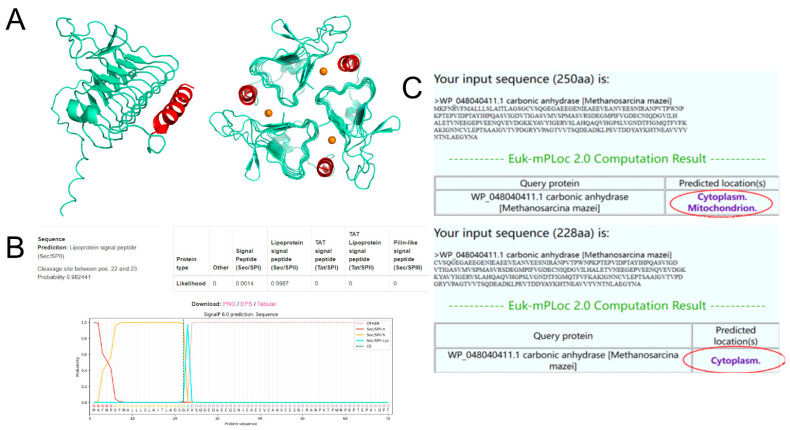
Bioinformatics analysis of MmaCA. (**A**) The predicted monomer (**left**) and trimeric structure (**right**) of MmaCA. Green: left-handed β-helix; Red: α-helix; Orange spheres: Zn²⁺ active centers at monomer interfaces. (**B**) Signal peptide sequence analysis identified a 22-amino-acid lipoprotein signal peptide at the MmaCA N-terminus. (**C**) Subcellular localization analysis, the red circle marks the predicted subcellular localization, full-length (**up**) MmaCA exhibited both cytoplasmic and mitochondrial targeting signals, truncated variant (**down**) retained only cytoplasmic localization.

The protein structure of MmaCA, predicted by AlphaFold, is depicted in Figure 2A. The left panel illustrates the monomeric structure of MmaCA, while the right panel displays its trimeric form, which shares structural similarity with the γ-CA derived from *M. thermophila*. The architecture of MmaCA is predominantly governed by β-strands, comprising seven complete turns that form a left-handed β-helix. Additionally, three Zn^2+^ active centers (represented as orange spheres) span the interface between monomers, coordinated by His residues provided by two distinct subunits. The red region in the figure highlights an α-helix composed of 17 amino acids (Lys-His-Thr-Asn-Glu-Ala-Val-Val-Tyr-Val-Asn-Thr-Asn-Leu-Ala-Glu-Gly). Given the presence of numerous hydrophobic amino acids within this structure, it is hypothesized that this α-helix may contribute to the intracellular localization of MmaCA. Consequently, we propose to truncate the sequence corresponding to this α-helix to enhance the secretion of MmaCA. We constructed a series of recombinant plasmids, pUAxp7166-mmaCAα1, α2, α3, and α4, featuring truncated α-helices of varying lengths (where α1, α2, α3, and α4 correspond to full truncation, 2/3 truncation, 1/2 truncation, and 1/3 truncation of the α-helix sequence, respectively), in an attempt to generate strains that would meet our objectives. Unfortunately, subsequent shake flask fermentation results over a 5 day period revealed that the enzymatic activity of all recombinant strains expressing the truncated MmaCA was indistinguishable from that of the parental Po1h strain. No statistically significant increase in CA activity was detected in either the intracellular or extracellular fractions compared to the negative control. This indicates that the hydrophobic α-helix, as a component of MmaCA, is likely crucial for the trimer self-assembly. Moreover, the β-helices forming the triangular prism’s lateral sides must dimerize to encapsulate Zn^2+^ ions, which is essential for the formation of the active site and manifestation of catalytic activity [54]. Therefore, truncating the sequences corresponding to the α-helical structure in MmaCA is deemed an undesirable strategy for achieving secretion.

- Attempt to promote the secretion of MmaCA by using LIP2 fusion protein

**Figure 3:**
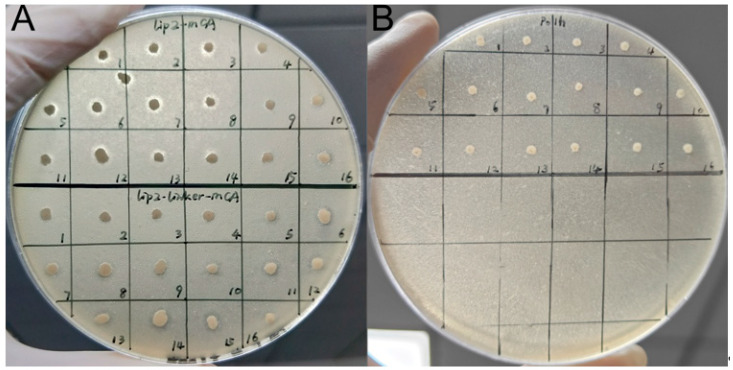
Detection of lipase activity in fusion proteins (16 replicates per group). (**A**) Two LIP2-MmaCA fusion designs. Top: Kex2 cleavage site-mediated connection between LIP2 and MmaCA. Bottom: Flexible linker-based linkage of LIP2 to MmaCA. (**B**) Initial strain Po1h.

Two distinct strategies were employed for the fusion of LIP2 [55] with MmaCA: one utilizing the Kex2 [56] protease cleavage site, and the other incorporating a flexible linker (G4S)_2_. Strain Po1h was employed as the host strain for heterologous expression of both fusion constructs. Functional evaluation was conducted via spot-plating on tributyrin-containing solid medium, with hydrolytic activity qualitatively assessed through observation of hydrolytic halo formation. However, fermentation experiments revealed that negligible carbonic anhydrase (CA) activity was detected in the recombinant strains’ supernatants. This demonstrated that while the LIP2-fusion strategy enabled extracellular secretion of the chimeric protein with preserved lipase activity (Figure 3), the fused MmaCA moiety failed to exhibit detectable CA functionality. We thought that this phenomenon paralleled our previous observations with α-helix truncation strategies, wherein both Kex2-processed MmaCA fragments and (G4S)_2_-linked MmaCA variants were incapable of forming the catalytically competent trimeric structure essential for CA activity (Figure 2A).

- Signal peptide prediction and subcellular localization analysis

The failure of both truncation strategies targeting the hydrophobic α-helix in MmaCA and N-terminal fusion with LIP2 to achieve extracellular secretion prompted investigation into intracellular retention mechanisms. SignalP 6.0 analysis [57] (Figure 2B) identified a 22-amino-acid lipoprotein signal peptide at the MmaCA N-terminus, potentially directing membrane localization. Subsequent subcellular localization prediction via Cell-PLoc 2.0 [58] (Figure 2C) revealed contrasting patterns: full-length MmaCA exhibited both cytoplasmic and mitochondrial targeting signals, while its signal peptide-truncated variant retained only cytoplasmic localization. This demonstrates that the lipoprotein signal peptide alone does not dictate intracellular retention, suggesting critical retention determinants reside in post-cleavage sequence elements downstream of the lipoprotein signal peptide.

Collectively, these findings indicate that MmaCA predominantly accumulates intracellularly, with previously detected extracellular activity likely arising from cell lysis artifacts. Subsequent efforts will focus on enhancing intracellular MmaCA expression in *Y. lipolytica* through promoter optimization, codon adaptation, and chaperone co-expression strategies.

#### 2.2.2. Optimize the Strength of the Heterozygous Promoter to Enhance the Expression Level of MmaCA

It is well established that promoter strength substantially influences protein expression levels. The plasmid employed in this study enables modular amplification of UAS1B copy numbers within the hp4d hybrid promoter through BioBrick assembly. This strategy generates enhanced hp4d derivatives (hp8d, hp12d, hp16d, hp20d, hp24d, hp28d, hp32d) through successive UAS1B duplications (Figure S1). Systematic evaluation of these variants will identify the optimal promoter configuration for MmaCA expression.

**Figure 4:**
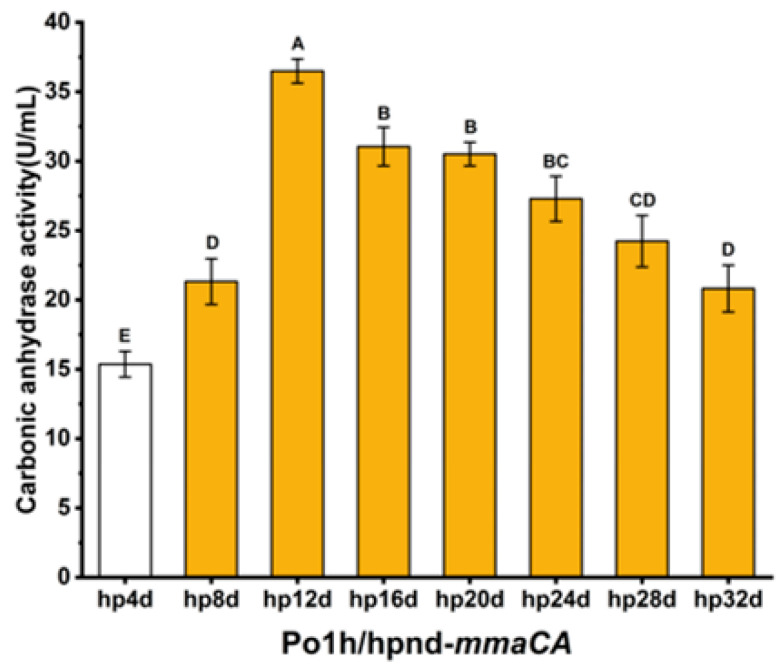
Optimization of the heterozygous promoter strength for *mmaCA*, hpnd represents the number of tandem UAS1B repeats contained in the promoter of the expression cassette.

The enzymatic activity assay results after shake-flask fermentation are shown in Figure 4. Intracellular enzyme activity of recombinant strains increased proportionally with UAS1B copy numbers ranging from 4 to 12. Notably, the strain carrying the hp12d hybrid promoter (12 UAS1B copies) exhibited peak intracellular enzyme activity at 36.5 ± 0.87 U/mL, representing a 2.40-fold enhancement compared to the conventional hp4d promoter-controlled strain (15.4 ± 0.9 U/mL). However, further amplification beyond 12 UAS1B copies resulted in gradual decline of catalytic performance. This demonstrates that copy number optimization of UAS1B elements effectively enhances MmaCA expression levels, with the hp12d promoter identified as the optimal regulatory configuration for MmaCA biosynthesis.

#### 2.2.3. Optimize the Gene Dosage to Enhance the Expression Level of MmaCA

Consistent with the principle of gene dosage effect on protein expression, multicopy plasmid systems constructed by BioBrick Assembly (Figure S2) were employed to integrate into the Po1h genome via SCOHR. Building upon the optimized hp12d promoter-driven *mmaCA* expression cassette, we constructed multicopy plasmids harboring 1–4 copies of the target cassette. Through two iterative rounds of site-specific integration at the preferred genomic locus, recombinant strains with 1–8 copy numbers were systematically generated for subsequent CA fermentation.

**Figure 5:**
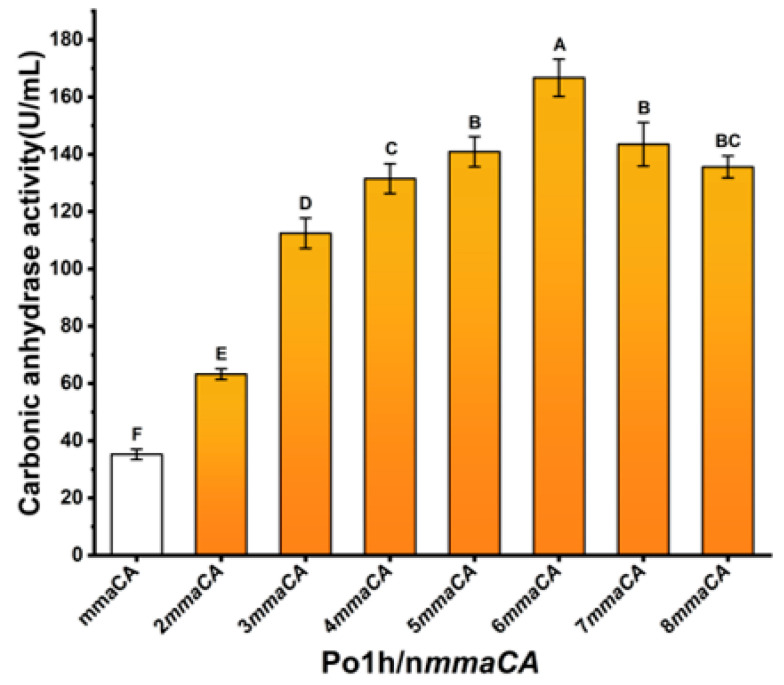
Optimization of the gene dosage for *mmaCA*; n*mmaCA* indicates the genomic copy number of the MmaCA expression cassette driven by the optimal hp12d promoter.

Recombinant strains cultured in BMSY medium under shake-flask conditions exhibited distinct carbonic anhydrase activity profiles as depicted in Figure 5. A positive correlation between *mmaCA* copy numbers (1–6) and enzymatic activity was observed, with catalytic efficiency showing proportional enhancement. Beyond 6 copies, a marked decline in enzymatic performance was recorded. The Po1h/6*mmaCA* strain achieved peak activity at 166.7 ± 6.5 U/mL, representing a 4.6-fold increase compared to the single-copy variant Po1h/1*mmaCA* (35.2 ± 1.8 U/mL). These findings demonstrate that strategic gene dosage optimization significantly enhances recombinant MmaCA enzymatic activity, with six copies being determined as the threshold for maximal enzymatic output.

#### 2.2.4. Optimize the Cultural Condition to Enhance the Expression Level of MmaCA

The cultivation conditions of microbial strains exert significant regulatory effects on both biomass accumulation and heterologous protein expression efficiency. To maximize the catalytic performance of MmaCA (Methanobactin metallochaperone carbonic anhydrase), we implemented a sequential single-factor optimization strategy targeting the 6-copy strain with highest enzymatic activity as the initial strain which included cultivation conditions such as the kind and concentration of carbon source, inoculum size, initial medium pH, and Zn^2+^ concentration.

**Figure 6:**
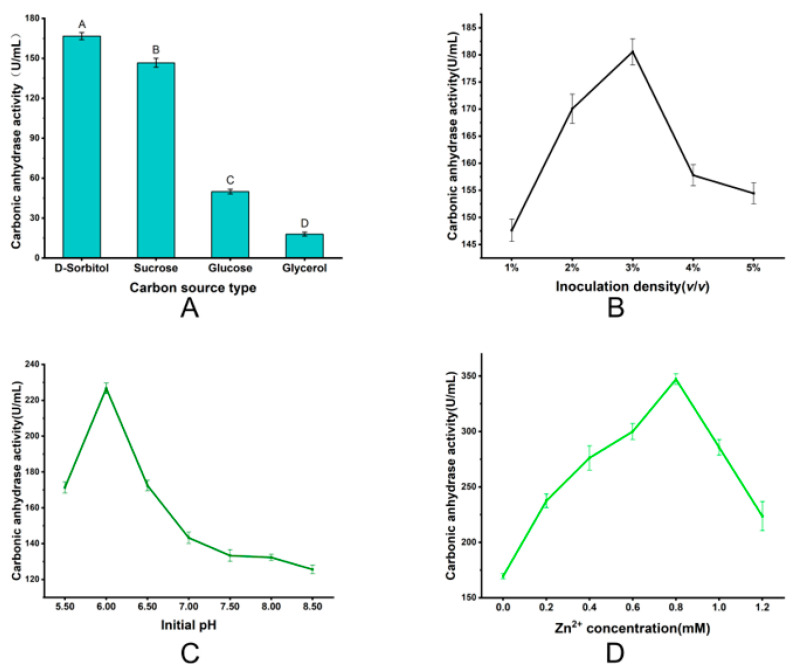
Optimization of the cultural condition for *mmaCA. *(**A**) Optimization of the carbon source. (**B**) Optimization of the inoculation density. (**C**) Optimization of the initial pH. (**D**) Optimization of the exogenous Zn^2+^ supplementation.

As shown in Figure 6A, the recombinant strain exhibited the highest yield of active MmaCA when using sorbitol as the carbon source, followed by sucrose, while glycerol and glucose showed relatively lower catalytic performance, indicating that the different carbon sources primarily influenced the expression level of the recombinant enzyme. Sorbitol concentration optimization revealed maximal enzyme activity at 5% (*m*/*v*) (167.9 ± 3.6 U/mL), with 7.8–9.9% reductions observed at suboptimal concentrations (6–7%) (Figure S3). Under these optimized parameters, 3% inoculation density yielded peak MmaCA activity (180.6 ± 2.4 U/mL) (Figure 6B). A subsequent initial pH experiment identified pH 6.00 as the optimal condition (226.7 ± 2.9 U/mL), enhancing activity by 1.8-fold compared to pH 8.5 (Figure 6C). Finally, exogenous Zn^2+^ supplementation significantly enhanced the enzymatic activity of MmaCA, with the maximum value (347.2 ± 4.8 U/mL) achieved at a concentration of 0.8 mM—representing a 2.05-fold increase compared to the Zn^2+^-free control (Figure 6D). Following comprehensive optimization of individual factors, fermentation of the recombinant strain was carried out with 5% sorbitol, 3% inoculum size, initial medium pH 6.00, and 0.8 mM Zn^2+^. The enzymatic activity assay results are shown in Figure S4. The MmaCA activity increased with prolonged cultivation time and peaked at 650 ± 16.7 U/mL on day 5. This represented a 3.89-fold improvement over the strain under unoptimized culture conditions and a remarkable 371.43-fold increase relative to the parental host strain Po1h.

### 2.3. High-Efficiency Heterologous Expression of SazCA in Y. lipolytica

Since SazCA is an exocrine protein, subsequent experiments focused on enhancing enzymatic activity. Following a similar approach to MmaCA, promoter length optimization experiments were first conducted, and gene dosage optimization was subsequently performed based on the experimental results. Further culture condition optimization was conducted in accordance with MmaCA. The results are shown in Figure S5.

Using the same strategy, we extended the promoter length and obtained corresponding recombinant strains Po1h/hpnd*-sazCA* (n = 4, 8, 12, …, 32). As shown in Figure S5A, when the UAS1B copy number ranged from 4 to 16, the enzymatic activity of the recombinant strains increased progressively with the increment of UAS1B copies. However, when the UAS1B copy number exceeded 16, the enzymatic activity decreased. Among these strains, the hp16d hybrid promoter recombinant strain containing 16 UAS1B copies exhibited the highest enzymatic activity of 56 ± 2.5 U/mL, which was 4.40-fold higher than that of the commonly used hp4d hybrid promoter recombinant strain (12.74 ± 0.85 U/mL). Similarly, optimizing the UAS1B copy number improved the expression level of *sazCA*, with hp16d identified as the optimal promoter. Subsequently, using the BioBrick Assembly method mentioned previously, recombinant plasmids *pU*Axp7166II-hp16d-n*sazCA* (n = 1–5) containing 1–5 copies of the *sazCA* gene cassettes were constructed. These plasmids were then used to generate recombinant strains Po1h/n*sazCA* (n = 1–5). The carbonic anhydrase activity in the fermentation supernatants of these strains is shown in Figure S5B. When the *sazCA* gene copy number ranged from 1 to 3, the carbonic anhydrase activity progressively increased with the increment of gene copies. At 3 copies, the activity reached a maximum of 244 ± 7.30 U/mL, which was 4.36-fold higher than that of the single-copy strain Po1h/1*sazCA* (56 ± 2.5 U/mL). However, when the gene copy number exceeded 3, the activity significantly decreased. Similarly, optimizing the gene dosage significantly enhanced the enzymatic activity of recombinant SazCA, with the optimal gene copy number identified as 3.

As shown in Figure S5C, the enzymatic activity of SazCA was evaluated under gradient Zn^2+^ concentrations ranging from 0 to 1.2 mM. The recombinant strain Po1h/3*sazCA* exhibited the highest enzymatic activity in the absence of Zn^2+^ addition (243.3 ± 12.81 U/mL). However, with increasing Zn^2+^ concentrations, the enzymatic activity of SazCA progressively decreased, indicating a dose-dependent inhibition by high concentrations of Zn^2+^ in this test. Among the tested carbon sources (sorbitol, sucrose, glucose, and glycerol), the recombinant strain exhibited the highest enzymatic activity in fermentation supernatant when sorbitol was used as the carbon source (243.75 ± 6.25 U/mL). In contrast, the enzymatic activity was significantly lower when sucrose, glycerol, or glucose served as carbon sources (Figure S5D). The enzymatic activity of SazCA reached its peak when the sorbitol concentration was 4% (271.85 ± 11.67 U/mL). Both lower and higher sorbitol concentrations resulted in reduced enzymatic activity (Figure S5E). The enzymatic activity of SazCA reaches its peak at an initial pH of 7 (285.19 ± 3.21 U/mL); both acidic and alkaline conditions will reduce it (Figure S5F). Enzymatic activity of SazCA reached the maximum when the inoculation density was 3% (328.57 ± 7.14 U/mL). Below this threshold, the enzymatic activity sharply declined, while exceeding this level resulted in a slight decrease (Figure S5G). Based on the comprehensive optimization of multiple single-factor conditions, the recombinant strain was subjected to fermentation (0 mM Zn^2+^, 4% sorbitol, initial medium pH 7.00, and 3% inoculation density). The enzymatic activity of SazCA increased progressively with fermentation duration, reaching a peak of 733.33 ± 16.67 U/mL on day 5, which was 3.00-fold higher than the strain under unoptimized culture conditions and a remarkable 419.05-fold increase relative to the parental host strain Po1h (Figure S5H).

### 2.4. SDS-PAGE, Purification, Mass Spectrometry and Glycosylation Identification

In parallel with the gene dosage optimization in previous experiments, the cell lysate supernatants of recombinant strains Po1h/n*mmaCA* were analyzed by SDS-PAGE. Similarly, the fermentation supernatants of recombinant strains Po1h/n*mmaCA* and Po1h/n*sazCA* were subjected to SDS-PAGE analysis, with results shown in Figures S6 and S7.

#### 2.4.1. SDS-PAGE of Supernatant

As shown in Figure S6A,B, the SDS-PAGE analysis revealed two distinct protein bands (marked by red arrows) within the molecular weight range of 25–35 kDa, corresponding closely to the theoretical size of MmaCA (27.5 kDa). The intensity of these target bands increased significantly when the gene copy number increased from 1 to 3. However, no further linear enhancement in band intensity was observed when the gene copy number increased from 4 to 6. Notably, lanes 7 and 8 (corresponding to 7 and 8 copies) still showed strong target protein bands, although their enzymatic activities were significantly lower than that of the 6-copy strain. This observation aligned with the enzymatic activity assay results up to 6 copies, indicating a positive correlation between gene dosage and functional enzyme production within this range. The strong bands observed in lanes 7 and 8 likely represent misfolded or inactive protein accumulated due to excessive metabolic burden caused by overexpression. Notably, an additional protein band of unknown origin (indicated by a blue arrow) emerged in the 35–45 kDa range, and its intensity increased with higher gene copy numbers. Mass spectrometry identification of this unknown band (Figure S6F) showed significant homology to multiple uncharacterized proteins in *Y. lipolytica*, with the highest matching score to YALI0E14190p. According to the NCBI database, the coding sequence (CDS) of YALI0E14190p shares similarity with the precursor mitochondrial malate dehydrogenase MDH1 of *Saccharomyces cerevisiae* (UniProt ID: P17505). The CDS region also encodes glyoxysomal and mitochondrial malate dehydrogenases (MDHs), which are involved in the tricarboxylic acid (TCA) cycle. This finding suggests a potential association between MmaCA (previously predicted as cytoplasmic or mitochondrial) and MDH-related metabolic pathways. Malate dehydrogenase participates in both the tricarboxylic acid (TCA) cycle and the glyoxylate cycle. The glyoxylate cycle plays a key role in the conversion of lipid-derived carbon sources into glucose precursors, particularly under conditions where cells rely on non-carbohydrate substrates. However, the sequence similarity of YALI0E14190p to malate dehydrogenases does not directly establish a functional or spatial link with MmaCA, and further experimental evidence is required to validate this potential association.

As shown in Figure S7A, SDS-PAGE analysis revealed a distinct protein band (marked by a red arrow) within the molecular weight range of 25–35 kDa, which corresponds to the theoretical size of SazCA (29.9 kDa). The intensity of this target band increased significantly when the gene copy number increased from 1 to 3, indicating enhanced protein expression. However, when the gene copy number exceeded 3, the band intensity decreased significantly. These results were fully consistent with the corresponding enzymatic activity assay results, indicating a positive correlation between gene dosage, protein expression level, and functional enzyme production within the optimal copy number range. This gene dosage effect, where expression peaks at a certain copy number and declines thereafter, is a well-documented phenomenon in heterologous protein expression systems.

#### 2.4.2. Purification, Mass Spectrometry and Glycosylation Identification

A His-tag was incorporated into the C-terminus of all CAs to facilitate protein purification. The SDS-PAGE results of MmaCA demonstrated that the purified protein in Lane 2 exhibited a single band within the 25–35 kDa range, consistent with its theoretical molecular weight (27.5 kDa). After concentration, the corresponding band in Lane 3 showed enhanced intensity (Figure S6C). The target protein band was excised from the gel and subjected to mass spectrometry (MS). As illustrated in Figure S6E, multiple peptide fragments matched the amino acid sequence of MmaCA derived from *M. mazei*, confirming the identity and purity of the recombinant protein.

As shown in Figure S7B, the SDS-PAGE analysis of purified SazCA revealed two closely spaced protein bands in Lane 1, both within the molecular weight range of 25–35 kDa. These bands closely matched the theoretical molecular weight of SazCA (29.9 kDa). MS of both bands (Figure S7C,D) demonstrated that multiple peptide fragments aligned with the amino acid sequence of SazCA derived from *S. azorense*, confirming the identity and purity of the recombinant protein.

Based on the aforementioned SDS-PAGE results showing double bands for both CAs, we hypothesized that this phenomenon might result from post-translational modifications (PTMs) of the proteins. To verify this, we performed deglycosylation assays to investigate potential glycosylation modifications. Results were shown in Figure S6D and Figure S7B. As shown in Endo H deglycosylation assays of the cell lysate supernatant from recombinant strain Po1h/6*mmaCA*, both target protein bands of MmaCA exhibited reduced molecular weights, ultimately aligning with the Endo H band (Figure S6D). For SazCA, the larger of the two protein bands showed diminished intensity, while the smaller band overlapped with the Endo H band and became indistinguishable (Figure S7B). These results indicate that N-linked glycosylation occurs in both intracellularly expressed MmaCA and extracellularly secreted SazCA.

### 2.5. Co-Expression of Auxiliary Proteins Can Significantly Enhance the Expression Levels of MmaCA and SazCA

Here, a co-expression strategy involving four auxiliary proteins—protein disulfide isomerase (Pdi), ER-resident protein (Kar2), ribosomal protein Rpl3, and *Vitreoscilla* hemoglobin (VHb)—to further enhance the production of MmaCA and SazCA was proposed. To ensure strain stability, the integration site was modified to the upstream region of the *Xpr2* gene, generating the plasmid pUXpr7166II (Figure 1A). Subsequently, the genes encoding the four auxiliary proteins (*vgb*, *pdi*, *kar2*, and *rpl3*) were ligated into the *Not* I-*Cla* I restriction sites, yielding four intracellular expression integration plasmids.

**Figure 7:**
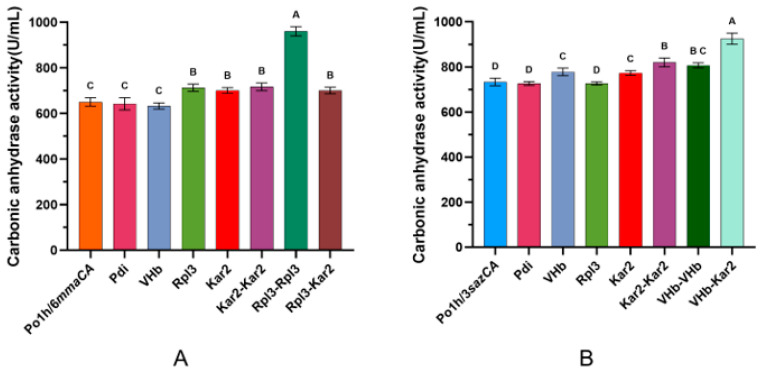
Enhancing carbonic anhydrase activity via co-expressed auxiliary proteins. (**A**) Results of MmaCA. (**B**) Results of SazCA.

The *Nsi* I-linearized plasmids were individually integrated into the previously optimized strains. Following 5-day shake-flask fermentation under the previously optimized conditions, enzyme activity of MmaCA and SazCA was measured, with results summarized in Figure 7. Compared to the parental strain Po1h/6*mmaCA*, recombinant strains co-expressing auxiliary proteins Pdi or VHb showed no statistically significant difference in enzyme activity. In contrast, strains co-expressing Rpl3 or Kar2 exhibited significant enhancement of activity. The engineered strains Po1h/6*mmaCA-rpl3* and Po1h/6*mmaCA-kar2* achieved enzyme activities of 712.5 ± 16 U/mL and 701 ± 12.7 U/mL, respectively, corresponding to 1.10-fold and 1.08-fold improvements over the control strain Po1h/6*mmaCA* (650 ± 18.4 U/mL). These results demonstrate that co-expression of Rpl3 or Kar2 effectively enhances MmaCA production (Figure 7A). On the other hand, the enzymatic activities of the recombinant strains co-expressing the auxiliary proteins Pdi and Rpl3 showed no significant differences, whereas significant enhancements were observed in strains co-expressing VHb (778 ± 17 U/mL) and Kar2 (772.7 ± 10.5 U/mL), which were 1.06-fold and 1.05-fold that of the parental strain Po1h/3*sazCA* (733 ± 17 U/mL). Overall, these findings indicate that co-expression of the auxiliary proteins VHb and Kar2 significantly enhances SazCA expression levels. Under the tested conditions, VHb exhibited a marginally stronger enhancing effect than Kar2 on average, though no statistically significant difference was observed between the two (Figure 7B).

As shown in Figure 7A, in the recombinant strains co-expressing Rpl3 and Kar2, as well as those expressing two copies of *kar2*, the enzymatic activity of MmaCA showed no significant difference compared to strains expressing Rpl3 or Kar2 alone. No synergistic effect was observed when these two auxiliary proteins were co-expressed. However, co-expression of two copies of *rpl3* significantly enhanced the enzymatic activity of MmaCA, reaching 960 ± 20 U/mL, which was 1.48-fold that of the initial strain. In comparison, the strain co-expressing VHb and Kar2 achieved a SazCA enzyme activity of 925 ± 25 U/mL, representing a 1.26-fold increase over the parental strain Po1h/3sazCA (733 ± 17 U/mL). Furthermore, it demonstrated a 1.19-fold and 1.20-fold increase relative to strains expressing VHb or Kar2 alone, respectively.

### 2.6. Mineralization Experiments of MmaCA and SazCA

To mitigate the potential interference of orotic acid [59,60] on mineralization processes, the finally optimized strains were subjected to *ura3* gene supplementation, yielding the strains used for mineralization, for the CA production. Fermentation supernatants (secretory SazCA) and cell lysate supernatants (intracellular MmaCA) were separately collected from shake-flask cultures, with corresponding supernatants from the initial strain Po1h (*ura3*^+^) serving as controls.

**Figure 8:**
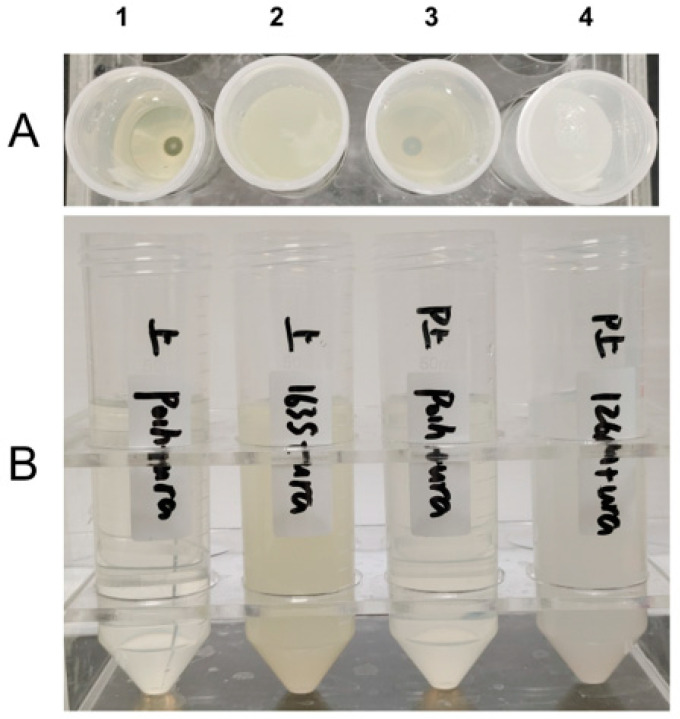
CO_2_ Mineralization Experiment. (**A**) Top view. (**B**) Front view. (**1**) Po1h fermentation supernatant. (**2**) SazCA strain fermentation supernatant. (**3**) Po1h lysate supernatant. (**4**) MmaCA strain lysate supernatant.

As illustrated in Figure 8, the control group 1 maintained a clear appearance without observable precipitation upon CaCl_2_ addition, whereas experimental group 2 exhibited rapid turbidity development. Control group 3 demonstrated only slight opacity, while experimental group 4 displayed immediate and pronounced precipitation formation. These findings collectively demonstrate that the MmaCA and SazCA produced by the engineered mineralization strains exhibit sufficiently high enzymatic activity to effectively drive rapid CaCO_3_ precipitation, underscoring their significant application potential in microbial-mediated mineralization technologies.

## 3. Discussion

Carbonic anhydrases are key enzymes for CO_2_ conversion, and their efficient production is the basis for their industrial application in CCS. In this study, we systematically investigated the heterologous expression of 10 CAs from different families in *Y. lipolytica*, and found that the expression levels of different CAs in *Y. lipolytica *were significantly different. Among them, MmaCA (γ-CA) and SazCA (α-CA) showed high expression potential, while the expression levels of β-CAs from different *Yarrowia* species were relatively low. This phenomenon may be related to the structure and characteristics of different CA families. α-CAs and γ-CAs have simple structures and are easy to fold correctly in heterologous hosts, while β-CAs usually exist in the form of dimers, tetramers, or octamers [61,62], and their correct assembly in *Y. lipolytica* may be restricted [59,63]. This limitation has been documented in cases of heterologous expression, such as the production of human IgG (a heterotetramer composed of two heavy chains and two light chains) in *Y. lipolytica*, where the majority of heavy and light chains fail to assemble correctly. Even under optimized signal peptide combinations, correctly assembled full-length IgG accounted for only 1.3% of the secreted products, while detectable functional assemblies represented merely 4.9% [64]. In addition, the codon preference of CA genes may also affect their expression in *Y. lipolytica*. Previous studies have shown that codon optimization according to the codon preference of *Y. lipolytica* can significantly improve the expression level of heterologous proteins [65,66], which may be a direction to improve the expression of β-CAs in future research.

Promoter engineering is an effective strategy to improve the heterologous expression level of proteins. The hp4d promoter is a commonly used hybrid promoter in *Y. lipolytica*, which is composed of 4 copies of UAS1B and the minimum promoter pLeumin [67]. UAS1B is an upstream activation sequence that can bind to transcription factors and promote gene transcription. In this study, we found that increasing the number of UAS1B copies can significantly improve the promoter strength and promote the expression of MmaCA and SazCA. However, when the number of UAS1B copies exceeds a certain threshold (12 copies for MmaCA and 16 copies for SazCA), the enzyme activity does not increase further, which may be due to the excessive binding of transcription factors to UAS1B, leading to the competition of transcription factors between the promoter and other endogenous genes, or the excessive burden on the host cells caused by the overexpression of the promoter [68]. This result is consistent with the previous research on the optimization of the hp4d promoter in *Y. lipolytica* [67], indicating that the optimization of UAS1B copies should be carried out according to the characteristics of the target gene.

Gene dosage is another important factor affecting the heterologous expression of proteins. Increasing the gene copy number can provide more templates for transcription, thereby improving the expression level of the target protein [68]. In this study, the functional enzyme activities of MmaCA and SazCA increased with the increase in gene copies within the optimal range, peaking at 6 copies for MmaCA and 3 copies for SazCA. However, when the gene copy number exceeded this threshold, the enzyme activity decreased significantly. This is because the excessive gene copies will increase the metabolic burden of the host cells, affect the normal growth and metabolism of the cells, and even lead to the instability of the genome [68]. Similar results have been reported in the heterologous expression of other proteins in* Y. lipolytica*. For example, Jiao et al. [53] found that the enzyme activity of the recombinant strain with 3 copies of *tll* gene was the highest, and the excessive copy number would reduce the enzyme activity. Therefore, the optimal gene copy number should be determined according to the characteristics of the target protein and the host strain.

The correct folding and assembly of recombinant proteins are crucial to their biological activity. When the target gene is overexpressed beyond the cellular folding capacity, a large number of nascent polypeptide chains may be incorrectly folded or assembled, leading to the accumulation of inactive protein aggregates and endoplasmic reticulum stress, which ultimately reduces the yield of functional enzyme [69]. Co-expression of auxiliary proteins involved in protein folding, transport, and synthesis can effectively solve this problem. In this study, we co-expressed four auxiliary proteins (Pdi, Kar2, Rpl3, VHb) and found that different auxiliary proteins have different effects on the expression of MmaCA and SazCA. Rpl3 (ribosomal protein) and Kar2 (endoplasmic reticulum resident protein) can improve the expression of MmaCA. Rpl3 can promote the synthesis of ribosomes and accelerate the translation rate of mRNA, and Kar2 can help the nascent polypeptide chains fold correctly, reducing the retention of incorrectly folded proteins in the endoplasmic reticulum [70]. VHb (*Vitreoscilla* hemoglobin) and Kar2 can improve the expression of SazCA. VHb can improve the oxygen uptake capacity of the host cells, promote the aerobic metabolism of the cells, and provide more energy for the synthesis and secretion of recombinant proteins [69]. However, the co-expression of Pdi (protein disulfide isomerase) had no significant effect on the expression of MmaCA and SazCA, which may be because the spatial structure of MmaCA and SazCA does not contain a large number of disulfide bonds, and the disulfide bond isomerization reaction is not a limiting factor for their folding [70]. In addition, the combined co-expression of VHb and Kar2 had a synergistic effect on the expression of SazCA, but the combined co-expression of Rpl3 and Kar2 had no synergistic effect on the expression of MmaCA. This may be related to the different expression characteristics of MmaCA and SazCA (MmaCA is intracellular expression, SazCA is extracellular secretion), and the different limiting factors of their expression processes.

The application potential of recombinant CAs in CO_2_ mineralization is the core goal of this study. CO_2_ mineralization is the process of converting CO_2_ into stable carbonate minerals, which is environmentally friendly and can realize the permanent sequestration of CO_2_ [71]. CAs can significantly accelerate the rate of CO_2_ hydration, thereby promoting the formation of carbonate minerals [2]. In this study, the CO_2_ mineralization experiment showed that the recombinant MmaCA and SazCA can significantly accelerate the formation of CaCO_3_ precipitation, which is consistent with the previous research results on the application of CAs in CO_2_ mineralization [34]. However, this study only carried out preliminary mineralization experiments under laboratory conditions, while the optimal conditions (such as temperature, pH, enzyme concentration, and Ca^2+^ concentration) for CO_2_ mineralization need to be further explored. In addition, the stability and reusability of recombinant CAs are also important factors affecting their industrial application. Future research can focus on the immobilization of recombinant CAs to improve their stability and reusability, and reduce the application cost [72].

This is the first systematic investigation of the heterologous expression of different families of CAs in *Y. lipolytica*, and two CAs with high expression potential were screened out, enriching the research on CA heterologous expression hosts. Multiple optimization strategies (promoter optimization, gene dosage adjustment, co-expression of auxiliary proteins) were combined to significantly improve the expression level of CAs, and the enzyme activity of SazCA reaches 925 U/mL. The comparison of carbonic anhydrase (CA) activities across different studies is inherently constrained by the absence of standardized assay protocols and unified calculation formulas. Taking an exemplary study on SazCA expression in *Escherichia coli* [34] as an example, based on the reported whole-cell activities of 8.355 U/mL/OD_600_ for intracellular expression and 11.43 U/mL/OD_600_ for surface-displayed enzyme, and assuming a typical OD_600_ value of 6 after 24 h of LB culture, the estimated volumetric activities would correspond to approximately 501.3 U/mL and 685.8 U/mL, respectively. These values are lower than the extracellular SazCA activity achieved in the present work (925 U/mL), indicating that the expression system developed herein exceeds the performance of most current heterologous CA production platforms.

Nevertheless, several limitations should be acknowledged in the present study. Only 10 CA genes were selected for expression screening; a broader panel of CA genes from diverse sources and families remains to be explored to further clarify the expression characteristics of different CAs in *Y. lipolytica*. The mechanism underlying the intracellular expression of MmaCA was not fully elucidated. The increased abundance of MDH in the recombinant strain suggests that MmaCA may be potentially associated with the MDH-related metabolic pathway. Notably, neither secretion signal peptide engineering nor fusion with a readily secreted protein succeeded in directing MmaCA to the extracellular space. This observation may be attributed to the strict microenvironmental requirements for MmaCA self-assembly. Although direct experimental evidence supporting this hypothesis is limited, our results imply that subcellular localization signals may exert a hierarchical priority in MmaCA targeting. Future work should focus on the intracellular localization and metabolic mechanism of MmaCA. In addition, CO_2_ mineralization assays were only performed at the shake-flask scale, and only the fundamental feasibility of recombinant CAs was verified herein. Further studies are warranted to evaluate the performance of recombinant CAs in large-scale CO_2_ mineralization using bioreactors, so as to validate their industrial application potential.

In summary, this study establishes *Y. lipolyticaas* a promising platform for the high-level production of functional microbial CAs, exemplified by SazCA and MmaCA. While sorbitol was identified as the optimal carbon source for maximizing yield under laboratory conditions, its economic feasibility for large-scale application warrants careful consideration. The choice of substrate ultimately presents a trade-off between yield and cost, a balance that must be evaluated with a specific application in mind. For high-value biocatalytic applications, a premium carbon source may be justifiable. However, for the targeted, cost-sensitive application in carbon capture and sequestration, future work must prioritize the use of low-cost or waste-derived feedstocks, such as crude glycerol or sucrose, to enable economically viable enzyme production at scale. Our optimized expression system provides the essential foundation for this crucial next step towards industrial implementation.

## 4. Materials and Methods

### 4.1. Strains, Plasmid, and Primers

The strains and plasmids used in this study are listed in Tables S1 and S2 (see Supplementary Materials for detailed information). *Y. lipolytica *Po1h (Ura^−^) was used as the host strain for heterologous expression. *E. coli* TOP10F’ was used for plasmid construction and propagation. The expression vector pUAxp7166 (a markerless integration vector with hp4d promoter, *xpr2* pre-signal peptide and terminator, and *ura3* selection marker) was preserved in our laboratory [53]. The restriction site was modified to generate pUAxp7166II for CA gene (include fusion CA genes) integration. Based on this, the integration site was further modified to XPR2 to generate pUXpr7166II for auxiliary protein integration. The CA genes (including *mmaCA*, *sazCA*, and 8 other CA genes, sequences could be found in Supplementary Materials) and primers used for plasmid construction and PCR verification were synthesized by Tsingke Biotechnology Co., Ltd. (Wuhan, China) and WuHan TianYi HuaYu Gene Technology Co., Ltd. (Wuhan, China) according to the sequences in the NCBI database. All plasmids integrate the target fragment between *Cla* I and *Sfi* I/*Not* I sites via restriction enzyme digestion and ligation or seamless cloning. The primer sequences are listed in Table S3 (see Supplementary Materials). The Phanta Max Master Mix for PCR amplification was obtained from Nanjing Vazyme Biotech Co., Ltd. (Nanjing, China) and the restriction enzymes were purchased from Thermo Fisher Scientific (China) Co., Ltd. (Shanghai, China) and TaKaRa Biomedical Technology (Beijing) Co., Ltd. (Beijing, China). The pEASY^®^-Basic Seamless Cloning and Assembly Kit, used for seamless cloning, and the DNA Ligation Kit Mighty Mix, used for ligation, were purchased from TransGen Biotech Co., Ltd. (Beijing, China) and TaKaRa Biomedical Technology (Beijing) Co., Ltd. (Beijing, China)., respectively. DNA fragment purification and plasmid extraction were performed using the FastPure^®^ Gel DNA Extraction Mini Kit (Vazyme, Nanjing, China) and the E.Z.N.A.^®^ Plasmid DNA Mini Kit I (Omega Bio-tek, Norcross, GA, USA), respectively. *E. coli* TOP10F’ was cultured at 37°C in LB medium (0.5% Yeast extract, 1% Tryptone, and 1% NaCl) containing 100 μg/mL ampicillin. All experimental procedures were performed according to the manufacturer’s instructions and standard molecular biology protocols.

### 4.2. BioBrick Assembly

A fixed pivot site (such as *Nsi* I or *Afl* II) and a pair of isocaudamers were used to finish the BioBrick Assembly. As an example, for the promoter optimization, after digesting the plasmid with* Nsi* I and *Bam* HI, recover the backbone. After digesting the plasmid with *Nsi* I and *Bgl* II, recover the 4UAS1B sequence. Ligate these two fragments to obtain a plasmid with the hp8d promoter. The 4UAS1B sequence flanked by *BamH* I/*Bgl* II isocaudamers can undergo copy number expansion via digestion-ligation cycles. Similarly, the expression box flanked by *Spe* I/*Nhe* I isocaudamers can undergo cassette copy number expansion via digestion-ligation cycles.

### 4.3. Markerless Integration of Y. lipolytica

The recombinant plasmids were linearized with restriction enzyme *Nsi* I and transformed into *Y. lipolytica* Po1h by the lithium acetate transformation method [73]. The transformed cells were inoculated into MD liquid medium (10% 10× YNB, 2% glucose and 0.2% 500× Biotin) for screening, then subcultured into fresh MD liquid medium 1.5 days after inoculation. Following visible turbidity, they were transferred to MO+Ura medium (10% 10× YNB, 2% oleic acid, 0.2% 500× Biotin and 0.02–0.1 g/L Uracil) for induction to eliminate the *ura3* selection marker.

### 4.4. Lipase Assay

A qualitative lipase assay was adopted to preliminarily verify the successful expression and extracellular secretion of fusion proteins. The detection medium was modified BMSY solid medium, containing basic BMSY components, 2% (*v*/*v*) tributyrin, 0.1% (*v*/*v*) Tween 80, and 15–20 g/L agar. Single colonies were inoculated onto the agar plate with a sterile toothpick. After static culture, the diameter of the hydrolysis halo around the inoculation site was observed; the size of the clear zone was positively correlated with extracellular lipase activity, which indirectly reflected the secretion level of target fusion proteins.

### 4.5. Shake-Flask Fermentation

The strain colony was inoculated into a 20 mL screw-cap vial containing 5 mL YPD liquid medium (1% yeast extract, 2% tryptone, 2% glucose). After 20 h of incubation, it was transferred to a 500 mL conical flask containing 50 mL BMSY medium (1% Yeast extract, 2% Tryptone, 5% Sorbitol), supplemented with 10% potassium phosphate buffer at pH 6.50. Prior to use, 5 mL of 10× YNB (134 g/L YNB) and 200 μL of 500× B (0.2 g/L biotin) were added to each flask, where the pH, carbon source and its concentration were adjusted according to experimental requirements. Incubation proceeded at 28 °C and 200 rpm. Unless otherwise specified, carbonic anhydrase activity was measured on day 5, as well as the CO_2_ mineralization experiment. All the experiments were performed with at least 3 biological replicates.

### 4.6. Sample Preparation for CA Activity Assay

For extracellular CA activity measurement, 1 mL of fermentation broth was centrifuged at 12,000 rpm for 2 min at 4 °C. The supernatant was carefully collected and stored on ice until use.

For intracellular CA activity measurement, the cell pellet obtained from the above centrifugation was washed twice with 1 mL of deionized water (12,000 rpm, 2 min each). The washed cells were resuspended in 250 μL of Breaking buffer (1.21% Tris base, 20% (*w*/*v*) glycerol, 0.1% 1M DTT, pH 8.0; 1 mM phenylmethylsulfonyl fluoride (PMSF) was added immediately before use), and an equal volume of 0.5 mm acid-washed glass beads was added. The mixture was vortexed at high speed for 1 min, followed by incubation on ice for 1 min. This cycle was repeated 20 times. After cell disruption, 750 μL of Breaking buffer was added, and the mixture was centrifuged at 12,000 rpm for 5 min at 4 °C. The resulting supernatant (intracellular supernatant) was collected for the activity assay.

### 4.7. Determination of CA Activity

The enzyme activity of carbonic anhydrase was determined using the electrode method (Wilbur-Anderson method), with slight modifications based on the protocol described by Li et al. [74]. Briefly, a 50 mL centrifuge tube was placed on ice, and 5 mL of barbital-KOH buffer and 0.5 mL of either boiled (inactivated) or unboiled crude enzyme sample (either cell lysate for intracellular CAs or fermentation supernatant for extracellular CAs) were added and mixed thoroughly. After recording the stable pH value of the mixture, 4.5 mL of CO_2_-saturated aqueous solution was rapidly but gently added, and the time required for the pH to decrease by 1 unit was immediately timed. The entire operation was performed under ice-bath conditions, and all solutions and samples were prepared and pre-cooled at 0–4 °C. The enzyme activity unit (U) was calculated using the formula: U = 10 × (T_0_/T_e_ − 1), where T_0_ and T_e_ represent the time required for the pH to drop by 1 unit in the presence of boiled (inactivated) and unboiled samples, respectively. Enzyme activity was expressed as units per milliliter of sample (U/mL).

### 4.8. CO_2_ Mineralization Experiment

CO_2_ mineralization assays were conducted following a modified protocol adapted from Giri et al. [75], with specific operational steps as follows: 2 mL of Tris-HCl buffer (1 M, pH 8.30) was added to 20 mL CO_2_-saturated aqueous solution, followed by gentle agitation at room temperature. Subsequently, 1 mL of prepared supernatant was introduced, and the mixture was allowed to stabilize for 2 min before injection into 10 mL of 2% CaCl_2_ solution. Precipitation profiles between experimental and control groups were visually compared.

### 4.9. Statistical Analysis

Data are presented as mean ± standard deviation (SD) from at least three independent experiments. One-way analysis of variance (ANOVA) followed by Tukey’s honestly significant difference (HSD) post hoc test for multiple group comparisons. Statistical significance was set at *p* < 0.05. The results of multiple comparisons are indicated by letter notation in the figures: groups sharing the same letter are not significantly different (*p* > 0.05), while groups with different letters are significantly different (*p* < 0.05).

## 5. Conclusions

In this study, we successfully realized the heterologous expression of 10 CAs from α, β, and γ families in *Y. lipolytica*, and screened two CAs with high expression potential (MmaCA and SazCA, belong to the α-CA and γ-CA families, respectively). Through the optimization of hybrid promoter strength, gene dosage, and co-expression of auxiliary proteins, the expression levels of MmaCA and SazCA were significantly improved. The intracellular enzyme activity of MmaCA reached 960 U/mL (64.42-fold higher than the initial strain), and the extracellular enzyme activity of SazCA reached 925 U/mL (70.08-fold higher than the initial strain). The CO_2_ mineralization experiment confirmed that both recombinant MmaCA and SazCA can efficiently accelerate the formation of CaCO_3_ precipitation, showing good application potential in CO_2_ capture and sequestration. This study establishes an efficient expression system of CAs in *Y. lipolytica*, provides a new strategy for the large-scale production of CAs, and lays a foundation for their industrial application in CO_2_ mineralization.

## Figures and Tables

**Table 1:** Information of CAs from different microbial sources.

Gene	Source	Accession Number	Gene Length/bp	Protein	Type
* pCA *	* Yarrowia lipolytica *	XP_505708.1	669	PCA	β
* bCA *	*Yarrowia* sp. B02	KAG5362900.1	813	BCA	β
* cCA *	*Yarrowia* sp. C11	KAG5359798.1	717	CCA	β
* mmaCA *	* Methanosarcina mazei *	WP_048040411	753	MmaCA	γ
* tauCA *	* Thermodesulfitimonas autotrophica *	WP_211328052.1	741	TauCA	γ
* cpCA *	*Cladocopium* sp.	MW759813.1	2847	CpCA	α
* dnCA *	*Durusdinium* sp.	MW759814.1	2829	DnCA	α
* cgiCA *	* Crassostrea gigas *	XM_011455522.3	927	CgiCA	α
* dsaCA *	* Dunaliella salina *	U53811.1	1611	DsaCA	α
* sazCA *	* Sulfurihydrogenibium azorense *	WP_012674680.1	765	SazCA	α

## Data Availability

Data are contained within the article and Supplementary Materials.

## Supplementary Materials

The following supporting information can be downloaded at https://www.mdpi.com/article/10.3390/ijms27104224/s1.

## Abbreviations

The following abbreviations are used in this manuscript:

CA	Carbonic anhydrase
CCS	Carbon capture and sequestration
CCM	CO_2_-concentrating mechanism
GRAS	Generally recognized as safe
BC	Biotin carboxylase
MDH	Mitochondrial malate dehydrogenases
SCOHR	Single Crossover Homologous Recombination
SDS-PAGE	Sodium dodecyl sulfate—polyacrylamide gel electrophoresis
UAS1B	Upstream activation sequences of *xpr2*
SD	Standard deviation
ANOVA	One-way analysis of variance
HSD	Tukey’s honestly significant difference
PMSF	phenylmethylsulfonyl
