# Supplementary material for: A Novel Strategy for Highly Efficient Heterologous Expression of Carbonic Anhydrase in Yarrowia lipolytica
Source: Int J Mol Sci. 2026 May 9;27(10):4224. doi: 10.3390/ijms27104224 (PMC13207147; doi:10.3390/ijms27104224)
Supplement: Supplementary file 1 [file ijms-27-04224-s001.zip › ijms-4268872-supplementary.pdf]

A Novel Strategy for Highly Efficient Heterologous Expression of Carbonic Anhydrase in *Yarrowia lipolytica*

Guowei Zhao†, Mengqin Zhu†, Liangcheng Jiao, Huanhuan Li, Yunchong Li, Kaixin Yang, Wenping Wei, Min Yang, Yunjun Yan *

Key Laboratory of Molecular Biophysics, the Ministry of Education; College of Life Science and Technology, Huazhong University of Science and Technology, Wuhan 430074, P. R. China

* Correspondence: yanyunjun@hust.edu.cn;

† These authors contributed equally to this work.

Supplementary Materials

The supplementary materials of this study include three summary tables for strains, plasmids, primer and gene sequences, agarose gel electrophoresis images for restriction digestion validation of plasmids constructed via Biobrick Assembly, SDS-PAGE gel images from the recombinant enzyme purification process and glycosylation identification assays, as well as the data from all optimization experiments.

1. Tables

Table S1. Strains in this study

Strain	Description	Source
Top10F′	For plasmid construction and proliferation	Laboratory storage
Po1h	MatA, ura3-302, xpr2-322, axp1-2, Ura-, ΔAEP, ΔAXP, Suc+	Laboratory storage
Po1h/hp4d-*pCA*	Po1h has integrated the plasmid pUAxp7166II-*pCA*	This work
Po1h/hp4d-*bCA*	Po1h has integrated the plasmid pUAxp7166II-*bCA*	This work
Po1h/hp4d-*cCA*	Po1h has integrated the plasmid pUAxp7166II-*cCA*，	This work
Po1h/hp4d-*mmaCA*	Po1h has integrated the plasmid pUAxp7166II-*mmaCA*	This work
Po1h/hp4d-*tauCA*	Po1h has integrated the plasmid pUAxp7166II-*tauCA*，	This work
Po1h/hp4d-*cpCA*	Po1h has integrated the plasmid pUAxp7166II-*cpCA*	This work
Po1h/hp4d-*dnCA*	Po1h has integrated the plasmid pUAxp7166II-*dnCA*	This work
Po1h/hp4d-*cgiCA*	Po1h has integrated the plasmid pUAxp7166II-*cgiCA*	This work
Po1h/hp4d-*dsaCA*	Po1h has integrated the plasmid pUAxp7166II-*dsaCA*	This work
Po1h/hp4d-*sazCA*	Po1h has integrated the plasmid pUAxp7166II-*sazCA*	This work
Po1h/*mmaCAα*1	Po1h has integrated the plasmid pUAxp7166II-*mCAα*1，	This work
Po1h/*mmaCAα*2	Po1h has integrated the plasmid pUAxp7166II-*mCAα*2，	This work
Po1h/*mmaCAα*3	Po1h has integrated the plasmid pUAxp7166II-*mCAα*3	This work
Po1h/*mmaCAα*4	Po1h has integrated the plasmid pUAxp7166II-*mCAα*4	This work
Po1h/*lip2-mmaCA*	Po1h has integrated the plasmid pUAxp7166II-*lip2-mmaCA*	This work
Po1h/*lip2-(G4S)$_2$-mmaCA*	Po1h has integrated the plasmid pUAxp7166II-*lip2-(G4S)$_2$-mmaCA*	This work
Po1h/hpnd-*mmaCA* (n = 8, 12, 16…32)	Po1h has integrated the plasmid pUAxp7166II-hpnd-*mmaCA* (n = 8, 12, 16…32)	This work
Po1h/n*mmaCA* (n = 1~4)	Po1h has integrated the plasmid pUAxp7166-hp12d-n*mmaCA* (n = 1~4)	This work
Po1h/n*mmaCA* (n = 5~8)	Po1h/4*mCA* has integrated the plasmid pUAxp7166-hp12d-n*mmaCA* (n = 1~4)	This work
Po1h/hpnd-*sazCA* (n = 4, 8, 12…32)	Po1h has integrated the plasmid pUAxp7166-hpnd-*sazCA* (n = 4, 8, 12…32)	This work

Po1h/n*sazCA* (n = 1~5)	Po1h has integrated the plasmid pUAxp7166-hp16d-n*sazCA* (n = 1~5)	This work
Po1h/6*mmaCA-pdi*	Po1h/6*mmaCA* has integrated the plasmid pUXpr7166-*pdi*	This work
Po1h/6*mmaCA-vgb*	Po1h/6*mmaCA* has integrated the plasmid pUXpr7166-*vgb*	This work
Po1h/6*mmaCA-kar2*	Po1h/6*mmaCA* has integrated the plasmid pUXpr7166-*kar2*	This work
Po1h/6*mmaCA-rpl3*	Po1h/6*mmaCA* has integrated the plasmid pUXpr7166-*rpl3*	This work
Po1h/6*mmaCA-rk*	Po1h/6*mmaCA-rpl3* has integrated the plasmid pUXpr7166-*kar2*	This work
Po1h/6*mmaCA*-2*rpl3*	Po1h/6*mmaCA-rpl3* has integrated the plasmid pUXpr7166-*rpl3*	This work
Po1h/6*mmaCA*-2*kar2*	Po1h/6*mmaCA-kar2* has integrated the plasmid pUXpr7166-*kar2*	
Po1h/3*sazCA-pdi*	Po1h/3*sazCA* has integrated the plasmid pUXpr7166-*pdi*	This work
Po1h/3*sazCA-vgb*	Po1h/3*sazCA* has integrated the plasmid pUXpr7166-*vgb*，	This work
Po1h/3*sazCA-kar2*	Po1h/3*sazCA* has integrated the plasmid pUXpr7166-*kar2*	This work
Po1h/3*sazCA-rpl3*	Po1h/3*sazCA* has integrated the plasmid pUXpr7166-*rpl3*	This work
Po1h/3*sazCA*-2*vgb*	Po1h/3*sazCA-vgb* has integrated the plasmid pUXpr7166-*vgb* ，	This work
Po1h/3*sazCA*-2*kar2*	Po1h/3*sazCA-kar2* has integrated the plasmid pUXpr7166-*kar2*	This work
Po1h/3*sazCA-vk*	Po1h/3*sazCA-vgb* has integrated the plasmid pUXpr7166-*kar2*	This work

Table S2. Plasmids in this study

Plasmid	Description	Source
pUC57-*bCA*	contain the CDS of *bCA*	Tsingke
pUC57-*cCA*	contain the CDS of *cCA*	Tsingke
pUC57-*mmaCA*	contain the CDS of *mmaCA*	Tsingke
pUC57-*tauCA*	contain the CDS of *tauCA*	Tsingke
pUC57-*cpCA*	contain the CDS of *cpCA*	Tsingke
pUC57-*dnCA*	contain the CDS of *dnCA*	Tsingke
pUC57-*cgiCA*	contain the CDS of *cgiCA*	Tsingke
pUC57-*dsaCA*	contain the CDS of *dsaCA*	Tsingke
pUC57-*sazCA*	contain the CDS of *sazCA*	TianYi HuaYu
pUAxp7166-*rol*	The markerless integration vector pUAxp7166 containing the *rol* gene, with the hp4d promoter and Xpr2 terminator, integrates into the sequence upstream of *axp1*	Laboratory storage
pUAxp7166II-*rol*	Modify the CDS sequence in pUAxp7166-*rol* to incorporate *Not* I and *Cla* I restriction sites flanking each cleavage site, serving as the template plasmid.	This work
pUXpr7166II-*rol*	Modify the upstream homologous sequence of *axp1* in pUAxp7166II-*rol* to the upstream homologous sequence of *xpr2*, serving as the template plasmid.	This work
pUAxp7166II-*pCA*	For integrating endogenous CA (*pCA*)	This work
pUAxp7166II-*bCA*	For integrating *bCA*	This work
pUAxp7166II-*cCA*	For integrating *cCA*	This work
pUAxp7166II-*mmaCA*	For integrating *mmaCA*	This work
pUAxp7166II-*tauCA*	For integrating *tauCA*	This work
pUAxp7166II-*cpCA*	For integrating *cpCA*	This work
pUAxp7166II-*dnCA*	For integrating *dnCA*	This work
pUAxp7166II-*cgiCA*	For integrating *cgiCA*	This work
pUAxp7166II-*dsaCA*	For integrating *dsaCA*	This work
pUAxp7166II-*sazCA*	For integrating *sazCA*	This work
pUAxp7166II-*mCAα*1	pUAxp7166II-*mmaCA* derivative, with the entire α-helical sequence truncated.	This work
pUAxp7166II-*mCAα*2	pUAxp7166II-*mmaCA* derivative, with the 2/3 α-helical sequence truncated.	This work
pUAxp7166II-*mCAα*3	pUAxp7166II-*mmaCA* derivative, with the half α-helical sequence truncated.	This work
pUAxp7166II-*mCAα*4	pUAxp7166II-*mmaCA* derivative, with the 1/3α-helical sequence truncated.	This work
pUAxp7166II-*lip2*-*mmaCA*	Containing *lip2* and *mmaCA* fusion genes, connected by kex2	This work
pUAxp7166II-*lip2*-*(G4S)*$_2$-*mmaCA*	Containing *lip2* and *mmaCA* fusion genes, connected by (G4S)$_2$	This work

pUAxp7166II-hpnd-*mmaCA* (n = 8, 12, 16…32)	Promoter optimisation	This work
pUAxp7166-hp12d-n*mmaCA*(n = 2, 3, 4)	Gene dosage adjustment	This work
pUAxp7166-hpnd-*sazCA* (n = 4, 8, 12…32)	Promoter optimisation	This work
pUAxp7166-n*sazCA* (n = 1~4)	Gene dosage adjustment	This work
pXprUra3	Resupply *ura3* to the upstream site of *xpr2*	This work
pUXpr7166II-*vgb*	Auxiliary Protein VHb Integration Vector	This work
pUXpr7166II-*pdi*	Auxiliary Protein Pdi Integration Vector	This work
pUXpr7166II-*kar2*	Auxiliary Protein Kar2 Integration Vector	This work
pUXpr7166II-*rpl3*	Auxiliary Protein Rpl3 Integration Vector	This work

Table S3. Primer list

Primer	Sequence (5'-3')	Restriction site	Purpose
lm-F	GGAAGATCTACtgatcaCGGGCAAAAGTGC	*Bgl* II	pUAxp7166II-*rol*
lm-R	GGCGGTAGCGAGCTTcaTGGTGGCGGCCGCTGTGGATGTGTGTGGTTG	*Not* I	
LEU-Kozak-ROL	CAACCACACACATCCACAGCGGCCGCCACCAtgAAGCTCGCTACCGCC	*Not* I	
ROL-ClaI-XPR2	GTGGGGACAGGCCATGGAATCGATttaCAGACAGGAGCCCTCGTTGAT	*Cla* I	
XT-F	TCCATGGCCTGTCCCCACG		
t-R	CTAGCTAGCGATAAGCTGTCAAACATGAGAATTC	*Nhe* I	
CrelipT-F	CTGCTGGAAGATGGCGATTAGC		pUXpr7166II-*rol*
lipT66-R	TACCGTTCGTATAATGTATGCTATACGAAGTTATCTCCACCTGTGTCAATCTTCTCAAG		
66upXPR2-F	ATAACTTCGTATAGCATACATTATACGAACGGTAGACAGTTAGAGCAGCAACGC		
upXPR2-MluI-R	GGGAGAGCTCTAGAGTCGACACGCGTCGACTAGTGGGCCCGTCGATGGGGTTTATATCG		
pCA-F1	CACGGCCGTTCTGGCCATGGCCCAGAAACCTCTTTTCC	*Sfi* I	pUAxp7166II-*pCA*
pCA-R1	ACAGGCCATGGAATCGATTTAATGCACAAGCTCGCCGG	*Cla* I	
bc-CAF	CACGGCCGTTCTGGCCATGGCTCAGAAGCCCCTGTTCC	*Sfi* I	pUAxp7166II-*bCA* & pUAxp7166II-*cCA*
bCA-R	ACAGGCCATGGAATCGATTTAGACAGGCATACCGAAGGTGGGGTC	*Cla* I	
cCA-R	ACAGGCCATGGAATCGATTTATCGCTGCTGGGGGGTGTCAAAGAC	*Cla* I	
mmaCA-F	CACGGCCGTTCTGGCCATGAAGTTCAACCGAGTCTTCATGGC	*Sfi* I	pUAxp7166II-*mmaCA*
mmaCA-R	ACAGGCCATGGAATCGATTTAGTGGTGGTGGTGGTGGTGGGCGTTGTATCCCTCGGCCAGGT	*Cla* I	
tauCA-F	CACGGCCGTTCTGGCCATGCTGACCGTCGTCGCCGTC	*Sfi* I	pUAxp7166II-*tauCA*
tauCA-R	ACAGGCCATGGAATCGATTTAGTGGGACTTGGTCTCACCGGTGGC	*Cla* I	
cpCA-F	CACGGCCGTTCTGGCCATGTTCGTCGCCATGTGGCGAG	*Sfi* I	pUAxp7166II-*cpCA*
cpCA-R	ACAGGCCATGGAATCGATTCACATTCGGTTGTAGGTGACTCGACCGA	*Cla* I	
dnCA-F	CACGGCCGTTCTGGCCATGTCCTCCATCGCCCGAGGC	*Sfi* I	pUAxp7166II-*dnCA*
dnCA-R	ACAGGCCATGGAATCGATTTAGGCTCGGTTGTAGGTGACTCGGC	*Cla* I	

cgiCA-F	CACGGCCGTTCTGGCCATGAAGTACCTGCTGGCCGTC	*Sfi* I	pUAxp7166II-*cgiCA*
cgiCA-R	ACAGGCCATGGAATCGATTTAGATGAAGACTCGGTGGATACACAGC	*Cla* I	
dsaCA-F	CACGGCCGTTCTGGCCATGGTCTCCGAGCCCCACGACTAC	*Sfi* I	pUAxp7166II-*dsaCA*
dsaCA-R	ACAGGCCATGGAATCGATTTAGGCGGCGGCGCCGTTGTA	*Cla* I	
sazCA-F	CTTTACTATTCTCACGGCCGTTCTGGCCGCCGAGGTCCACCACTGG	*Sfi* I	pUAxp7166II-*sazCA*
sazCA-R	GTGGGGACAGGCCATGGAATCGATTTAATGGTGATGGTGATGGTGGTTG	*Cla* I	
ClaI-m-αR1	GTGGGGACAGGCCATGGAATCGATTTAGGCGTTGTAGTAGGCGTAGTCGTCGGTAACC	*Cla* I	α-helical sequence truncated plasmids
ClaI-m-αR2	GTGGGGACAGGCCATGGAATCGATTTAGGCGTTGTAAGCCTCGTTGGTGTGCTTG	*Cla* I	
ClaI-m-αR3	GTGGGGACAGGCCATGGAATCGATTTAGGCGTTGTAGTAGACGACAGCCTCGTTGGT	*Cla* I	
ClaI-m-αR4	GTGGGGACAGGCCATGGAATCGATTTAGGCGTTGTAGGTGTTAACGTAGACGACAGCCT	*Cla* I	
rhlip2-F	CAACCACACACATCCACAGCGGCCGCCACCATGAAGCTTTCCACCATCCTCTTCAC	*Not* I	pUAxp7166II-*lip2-mmaCA* & pUAxp7166II-*lip2-(G4S)$_2$-mCA*
rhlip2-R	CCATGAAGACTCGGTTGAACTTCATTCGCTTGATACCACAGACACCCTCGG		
rhmCA-F	CCGAGGGTGTCTGTGGTATCAAGCGAATGAAGTTCAACCGAGTCTTCATGG		
rhmCA-R	AACGTGGGGACAGGCCATGGAATCGATTTAGGCGTTGTATCCCTCGGCC	*Cla* I	
rhmCA-2GS-F	GGTGGAGGTGGCTCTGGAGGAGGCGGCTCCATGAAGTTCAACCGAGTCTTCATGG		
rhlip2-2GS-R	GGAGCCGCCTCCTCCAGAGCCACCTCCACCGATACCACAGACACCCTCGG		
Vgb-NotI-F	CAACCACACACATCCACAGCGGCCGCCACCATGTTGGATCAACAGACCATTAACATC		pUXpr7166II-*vgb*
Vgb-ClaI-R	GTGGGGACAGGCCATGGAATCGATCTATTCAACAGCTTGAGCGTACAAATCTG		
Pdi-NotI-F	CAACCACACACATCCACAGCGGCCGCCACCATGAAGTTCACTGCCCTCAC		pUXpr7166II-*pdi*
Pdi-ClaI-R	GTGGGGACAGGCCATGGAATCGATTTAAAGCTCATCATCAATCTTG		
Rpl3-NotI-F	CAACCACACACATCCACAGCGGCCGCCACCATGTCCCACCGAAAGTACGAAC		pUXpr7166II-*rpl*
Rpl3-ClaI-R	GTGGGGACAGGCCATGGAATCGATTTAAAGCTTAAAGGTCCTTCTTGAGAG		
Kar2-NotI-F	CAACCACACACATCCACAGCGGCCGCCACCATGAAGTTCTCTATGCCTTCGTGG		pUXpr7166II-*kar2*

Kar2-ClaI-R	GTGGGGACAGGCCATGGAATCGATTTAAAGCTCATCGTGGAAAGGAGCC	
upXpr2Ura-F	CCGAGAAACAGGCCTTTGTCagatctGACAGTTAGAGCAGCAACGCG	
upXpr2-NsiI-R	CTATAAAAATAGGCGTATCACGGGCCCATGCATAGGACCCCGGATCG	
upXpr2Amp-F	CGATCCGGGGTCCTATGCATGGGCCCGTGATACGCCTATTTTTATAG	
OriupXpr2-R	CAATCAATGTTTCAGGGATGCATACATATGCGGTGTGAAATACCGCAC	pXprUra3
upXpr2-NsiI-F	GTGCGGTATTTCACACCGCATATGTATGCATCCCTGAAACATTGATTG	
upXpr2Ura-R	GTTGTGGGGGGTAATTGGATTGAGGGCCCGTCGATGGGGTTTATATC	
Ura3-F	TCAATCCAATTACCCCCCACAAC	
Ura3-R	GACAAAGGCCTGTTTCTCGG	

The underlined parts represent the restriction sites, or the homologous arms used for SOE PCR or seamless cloning.

2. Figures

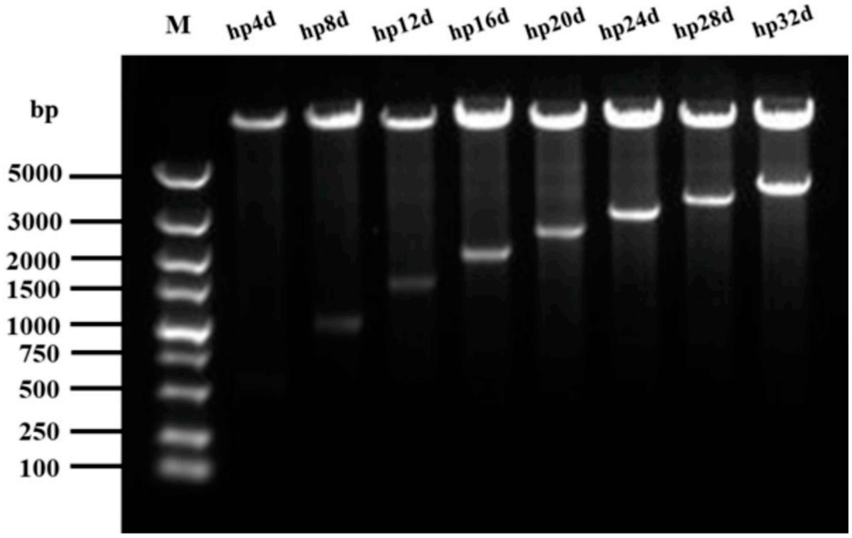

Figure S1. Enzyme digestion validation of heterozygous promoters (*Bgl* II&*Mlu* I)

M: DNA Marker; **Lane names** correspond to the respective heterozygous promoters.

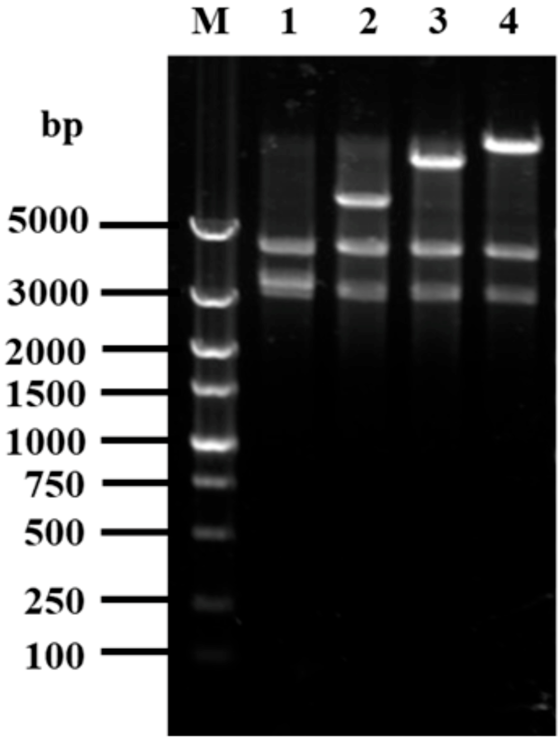

Figure S2. Enzyme digestion validation of multicopy plasmids (*Spe* I & *Bgl* II & *Afl* II)

M: DNA Marker; **Lane names** correspond to the respective gene copy number.

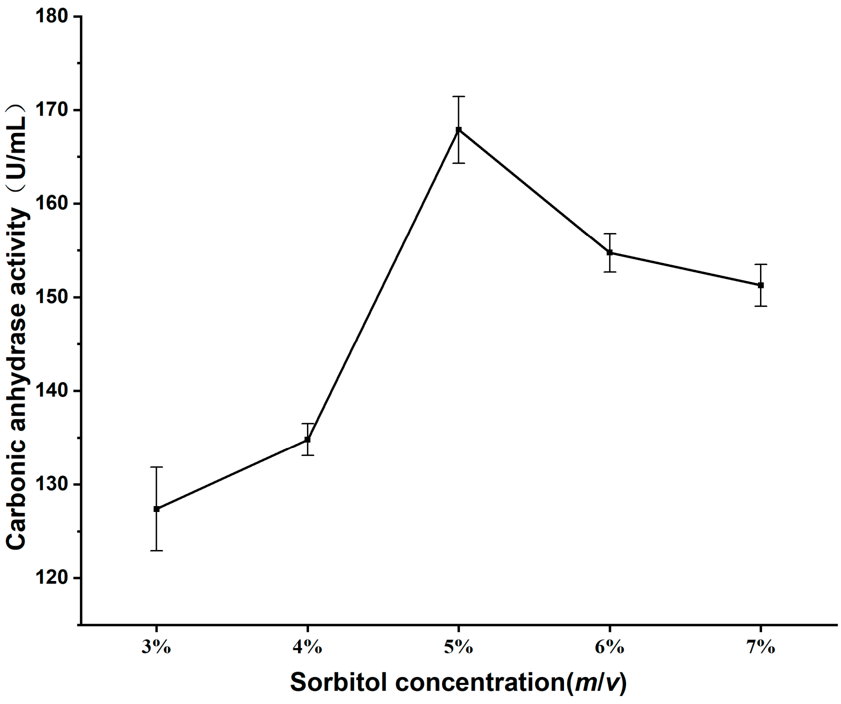

Figure S3. Optimization of sorbitol concentration for *mmaCA*

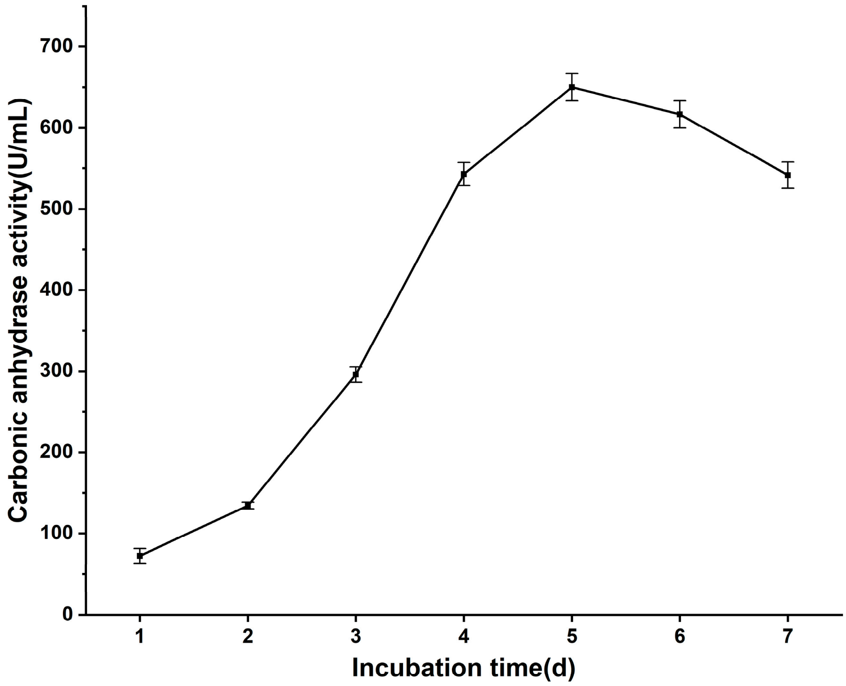

Figure S4. Optimization of the incubation time for *mmaCA*

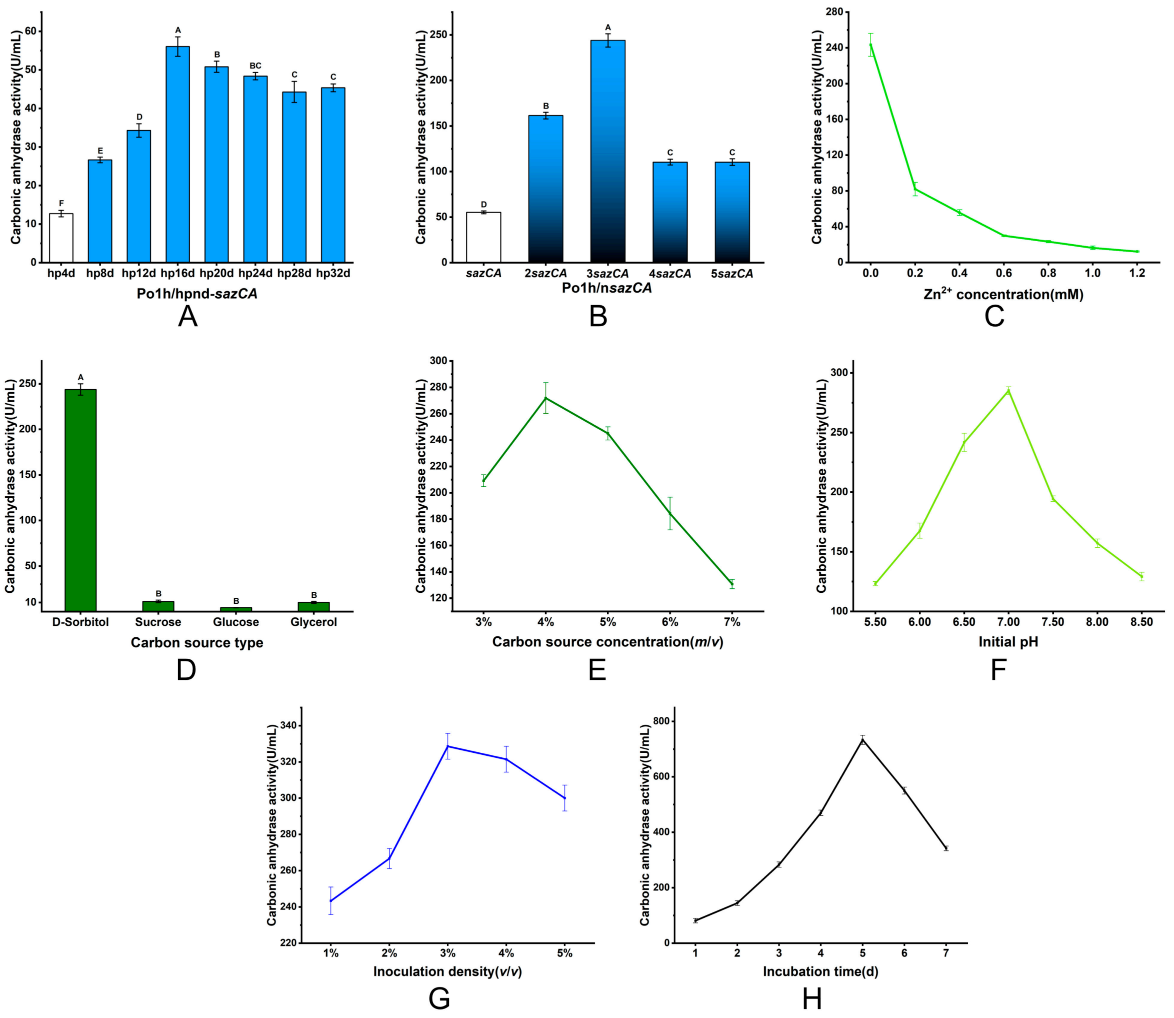

Figure S5. Sequential single-factor optimization for *sazCA*

A: Optimization of the heterozygous promoter strength; hpnd represents the number of tandem UAS1B repeats contained in the promoter of the expression cassette.

B: Optimization of the gene dosage; n*sazCA* indicates the genomic copy number of the SazCA expression cassette driven by the optimal hp16d promoter.

C: Optimization of the the exogenous Zn^{2+} supplementation;

D: Optimization of the carbon source; **E:** Optimization of the sorbitol concentration;

F: Optimization of the initial pH; **G:** Optimization of the inoculation density;

H: Optimization of the incubation time.

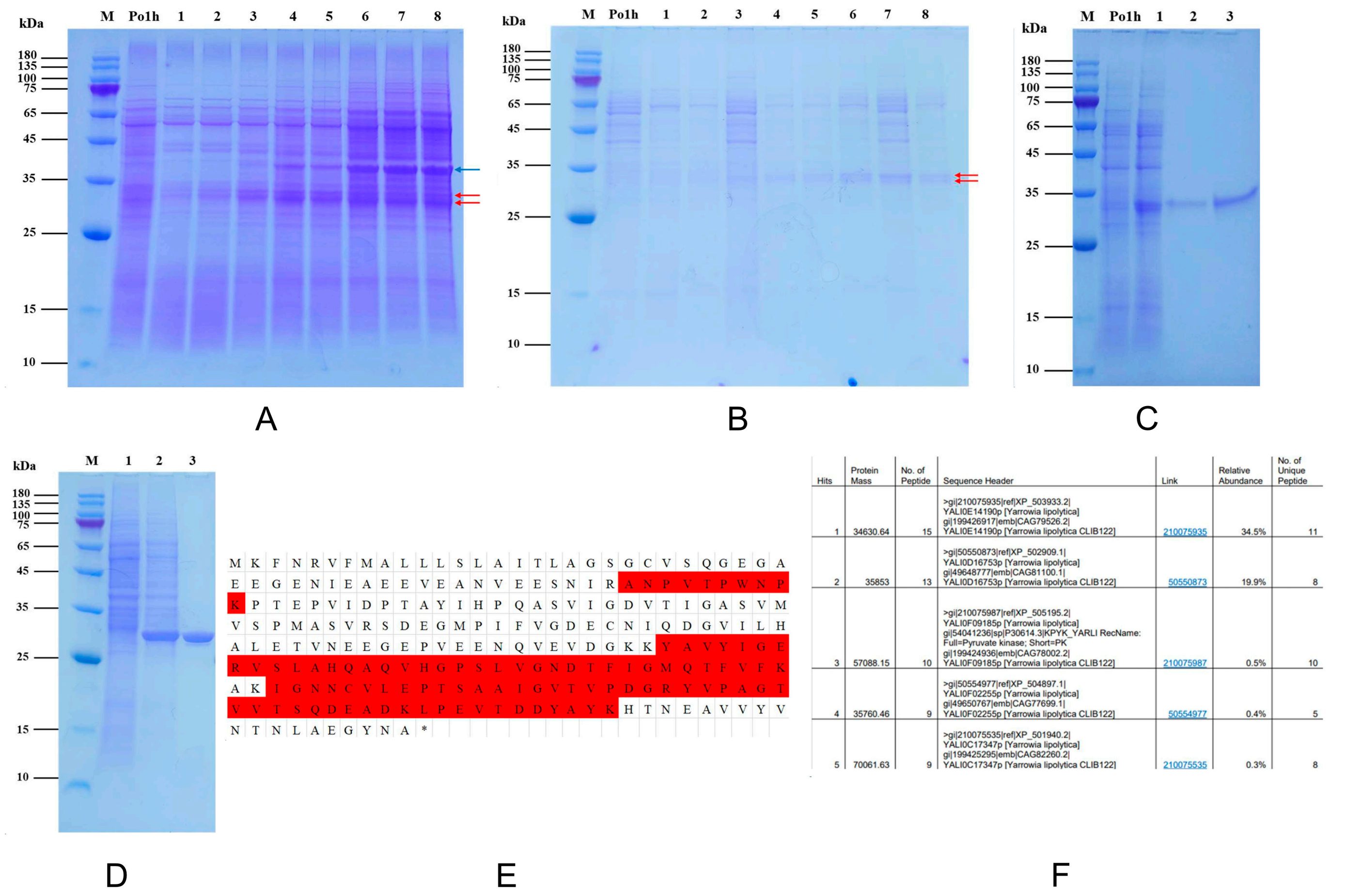

Figure S6. Identification of MmaCA

A: SDS-PAGE analysis of supernatant from lysed recombinant strain Po1h/n*mmaCA* (n = 1 - 8), M: Pre-stained protein molecular weight marker, lane 1 - 8: Po1h/n*mmaCA* (n = 1 - 8), the red arrows indicate the target MmaCA protein bands, which are clearly separated from other non-specific bands, the blue arrow indicates the unknown protein band referred to in F;

B: SDS-PAGE analysis of fermentation supernatants from recombinant strain Po1h/n*mmaCA* (n = 1 - 8), M: Pre-stained protein molecular weight marker, lane 1 - 8: Po1h/n*mmaCA* (n = 1 - 8), the red arrows indicate the target MmaCA protein bands, which are clearly separated from other non-specific bands;

C: SDS-PAGE results of purified MmaCA, M: Pre-stained protein molecular weight marker, lane 1: Lysate supernatant from Po1h/6mCA, lane 2: Lysate supernatant after purification; lane 3: Purified supernatant after concentration.

D: Deglycosylation analysis of the lysate from recombinant strain Po1h/6*mma*CA, M: Pre-stained protein molecular weight marker, lane 1: Po1h/6mCA lysate, lane 2: Lysate treated with Endo H for deglycosylation, lane 3: Endo H;

E: Mass spectrometry identification results for the MmaCA recombinant protein (red regions indicate successfully matched amino acid sequences);

F: Analysis of mass spectrometry alignment results for unknown protein bands.

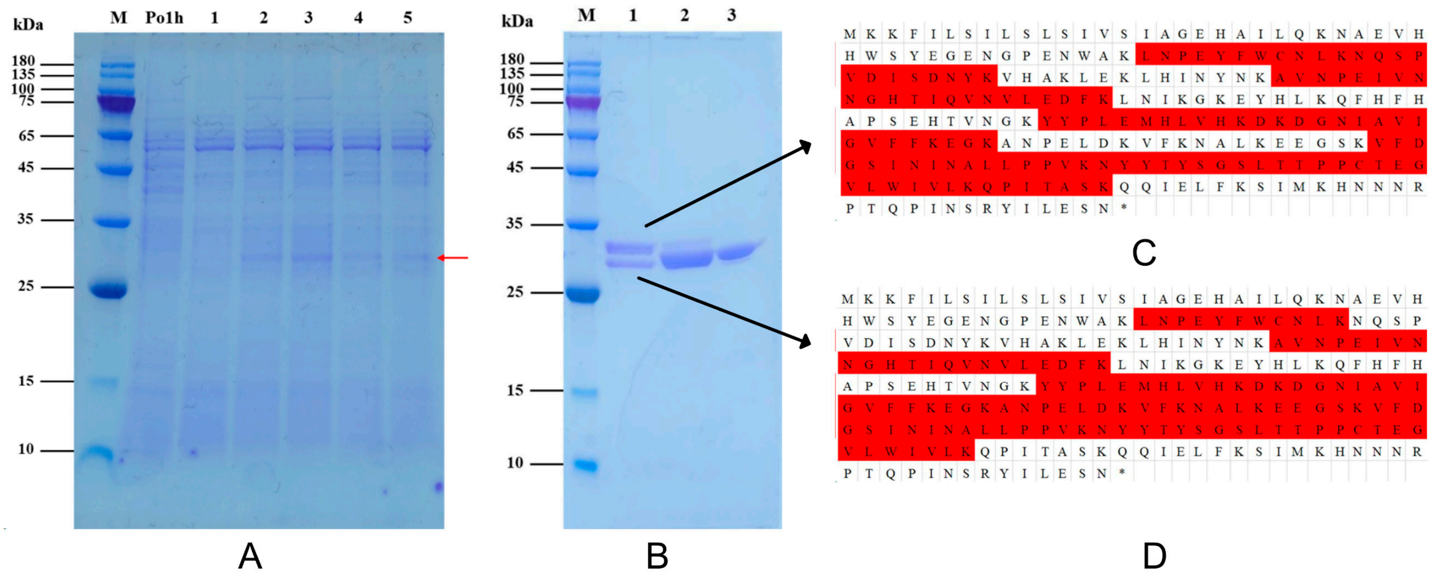

Figure S7. Identification of SazCA

A: SDS-PAGE analysis of fermentation supernatants from recombinant strain Po1h/n*sazCA* (n = 1 - 5)

B: Purification and deglycosylation identification of fermentation supernatant from recombinant strain Po1h/3*sazCA*, M: Pre-stained protein molecular weight marker, lane1: Po1h/3sazCA purified supernatant, lane 2: Purified supernatant treated with Endo H for deglycosylation, lane 3: Endo H;

C, D: Mass spectrometry identification results of recombinant SazCA (red regions denote successfully matched amino acid sequences), **C** corresponds to the higher-molecular-weight protein band in the SazCA-purified supernatant, **D** corresponds to the lower-molecular-weight protein band.

3. Gene Sequences

***pCA*:**

ATGGCCCAGAAACCTCTTTTCCGATTCGGAGCCACCGACTCGCTCGCCACAT
TGCTGGAGCGAAACGAAAAATGGGCCAACCGAGTGTCATCAGTGAGACCCTCG
CTGTTCCCCACAAACGCTCAGGGCCAGGCCCCCAAGATTCTATGGATCGGCTGC
TCCGACTCACGAGCCGGAGAAGGCTGTCTGGATCTGCTGCCTGGAGAGGTGTTT
GTGCACAGAAACATTGCCAACTTGCTGCCCGACTCTGACTTCTCGTCGCTCTCAG
TCATCCAGTTTGCCGTCCAGGTTCTCAAGGTGCGACACATCATTGTCTGCGGTCA
CTACGACTGCGGAGGAGTGTGGTCGTCGCTCACCTCCAAGAAGCTGGGTATCAT
TGACCACTGGCTGCGACCCATTCGAGACACAAAGGTGCGACATAAGGCCATGCT
GGATGCCATCGAGGACCCCAAGGACAAGTGTGCCCGCCTCGTGGAGCTCAACGT
GTGCGCCCAGGTCAACAATCTCAAGCGAAACACTGTGATCATCGAGGCCCAGG
GTGAGCGAGATCTCCAGATCCACGGCGTCGTCTACGACCCCGGAAGCGGTCTGC
TCAAGGAGATTATGGTCCCCGAAGACGAGTATGCTGAGGACTACTTCGTGTCCG
ACGCCGGCGAGCTTGTGCATTAA

***bCA*:**

ATGGCTCAGAAGCCCCTGTTCCGATTCGGCGCTACCGACTCTCTGGCTACCC
TGCTGGAGCGAAACGAGAAGTGGGCTAACCGAGTCTCCTCCGTCCGACCTTCTC
TGTTCCCTACCAACGCCCAGGGTCAGGCTCCTAAGATCCTGTGGATCGGATGCTC
TGACTCCCGAGCTGGTGAGGGTTGTCTGGACCTGCTGCCTGGTGAGGTTTTCGTC
CACCGAAACATCGCCAACCTGCTGCCTGACTCTGACTTCTCCTCCCTCTCCGTCA
TTCAGTTCGCCGTCCAGGTGCTGAAGGTTCGACACATTATCGTCTGCGGCCACTA
CGACTGCGGCGGTGTTTGGTCTTCTCTCACCTCCAAGAAGCTGGGCATCATCGAC
CACTGGCTGCGACCTATTCGAGACACCAAGGTCCGACACAAGGCCATGCTGGAC
GCTATTGAGGACCCCAAGGACAAGTGCGCCCGACTGGTTGAGCTGAACATTTGT
GCCCAGGTGAACAACCTGAAGCGAAACACCGTGATCATCGAGGCCCAGGGAGA
GCGAGACCTGCAGATCCACGGTGTTGTGTACGACCCCGGTTCCGGTATGCTGAA
GGAGATCAGCGTCCCCGACGACGACTCTTGTACCAACGCTTCCTACGTCGCCATT
GACAACATTCACTCCAAGCGATCCGGCATCAAGACCCCTCAGCAGATGACCCCT
CACCAGCACGGTCGATTCATCGTTGCCGAGGCTTCCGCTGTCGACGACCGACTG
CACGGTGTTCTGTTCCTCGCTGCTAAGGACCCCACCTTCGGTATGCCTGTC

cCA:

ATGGCTCAGAAGCCCCTGTTCCGATTCGGCGCTACCGACTCTCTGGCTACCC
TGCTGGAGCGAAACGAGAAGTGGGCTAACCGAGTCTCCTCCGTCCGACCTTCTC
TGTTCCCTACCAACGCCCAGGGTCAGGCTCCTAAGATCCTGTGGATCGGATGCTC
TGACTCCCGAGCTGGTGAGGGTTGTCTGGACCTGCTGCCTGGTGAGGTTTTCGTC
CACCGAAACATCGCCAACCTGCTGCCTGACTCTGACTTCTCCTCCCTCTCCGTCA
TTCAGTTCGCCGTCCAGGTGCTGAAGGTTCGACACATTATCGTCTGCGGCCACTA
CGACTGCGGCGGTGTTTGGTCTTCTCTCACCTCCAAGAAGCTGGGCATCATCGAC
CACTGGCTGCGACCTATTCGAGACACCAAGGTCCGACACAAGGCCCTGCTGGAC
GCTATTGAGGACCCTAAGGACAAGTGTGCCCGACTCGTCGAGCTGAACGTTTGT
GCTCAGGTGAACAACCTCAAGCGAAACACCGTGATCATCGAGGCCCAGGGAGA
GCGAGACCTGCAGATCCACGGTGTTGTCTACGACCCCGGTTCCGGTCTGCTGAA
GGAGATTTCCGTCCCCGAGGACGAGTACGCTGAGGACTACTTCGTCTGCTCCTCC
ATCCTGGGTTGGAACCTGAACACCAACTCTAAGCGATCCGTCTTTGACACCCCCC
AGCAGCGATAA

mmaCA:

ATGAAGTTCAACCGAGTCTTCATGGCCCTGCTGCTGTCCCTGGCTATCACCC
TGGCTGGTTCTGGTTGCGTCTCCCAGGGTGAGGGTGCTGAGGAGGGTGAGAACA
TCGAGGCTGAGGAGGTCGAGGCTAACGTCGAGGAGTCTAACATTCGAGCTAACC
CCGTCACCCCCTGGAACCCTAAGCCTACCGAGCCTGTTATTGACCCCACCGCTTA
CATTCACCCCCAGGCTTCTGTTATTGGAGACGTCACCATCGGCGCCTCCGTTATG
GTTTCCCCTATGGCTTCCGTCCGATCCGACGAGGGTATGCCTATTTTCGTCGGCG
ACGAGTGCAACATCCAGGACGGTGTTATTCTGCACGCCCTCGAGACCGTTAACG
AGGAGGGTGAGCCTGTCGAGGAGAACCAGGTTGAGGTCGACGGTAAGAAGTAC
GCCGTGTACATCGGCGAGCGAGTCTCTCTGGCTCACCAGGCTCAGGTTCACGGA
CCTTCTCTGGTGGGTAACGACACCTTCATTGGAATGCAGACCTTTGTTTTTAAGG
CCAAGATCGGTAACAACTGCGTTCTGGAGCCCACCTCCGCTGCTATTGGTGTTAC
CGTCCCCGACGGTCGATACGTTCCTGCTGGTACCGTCGTCACCTCCCAGGACGA
GGCTGACAAGCTGCCTGAGGTTACCGACGACTACGCCTACAAGCACACCAACG
AGGCTGTCGTCTACGTTAACACCAACCTGGCCGAGGGATACAACGCCCACCACC
ACCACCACCACTAA

***tauCA*:**

ATGCTGACCGTCGTCGCCGTCGGCGCCACCCTGTGTTTCACCGCCGGCTGCG
CCTCCACCCAGACCACCGCTACCAAGGAGCCCGCCAAGCCCGCCAACATCCGA
CCCAACGTCGTCACCACCTTCAACCCCACCACCGAGACCCCCGTCATCGCCAAG
GACGCCTACATCGACCCCCTCGCCTCCGTCATCGGCAACGTCGAGATCGGCTCC
AAGGTCTACGTCGCCCCTTTCGCCTCCGTCCGAGGCGACGAGGGTCAGCCTATTT
ACGTTGGCGAGGGTTCCAACGTGCAGGACGGTGTCGTTCTGCACGCCCTGGAGA
CCGAGGACAACGGCAAGCCCGTCGAGAAGAACCTCGTCGAGTACGGAGGCAAG
AAGTACGCCGTGTACATTGGTAAGCACGTCTCTCTCGCCCACCAGGCCCAGGTC
CACGGCCCTGCTCTGGTGGACGACGGAACCTTCGTGGGCATGCAGGCCCTGGTC
TTTAAGGCCCAGGTCGGTAAGAACTGCGTGATCGAGCCCGGCGCCAAGCTGCTC
AACGGCGTGAAGGTCCCCGACGGCCGATACGTGCCCGCCGGTACTGTGGTCACC
ACCCAGGCCCAGGCCGACAAGCTGCCCGTGATCACCGACGCCTACCCCCTGAA
GAACCTCAACAAGGGCGTCCTGCACGTCAACGAGCAGCTCGCCGAGGGTTACCT
GAAGGCCCAGGAGGGAGCCACCGGTGAGACCAAGTCCCACTAA

***cpCA*:**

ATGTTCGTCGCCATGTGGCGAGCCCTGGTCGGCGTCTGCGTCGCTGCTTCCT
CCGCCGAGACCGGCCGACGACTGCAGAACTACGGCGGCTGCCCCCAGTGGCAG
AACACCACCTTCTCCGACGAGAAGTCCCCCTTTGGTTGGCCTCTGACCCCTAACG
ACTGGTCCTTCTCCGAGCAGCGACTGTGGCCCGAGTCCTACCTGGCCTGTGGCGG
TCTGCGACAGTCCCCCATCGACGTCCCCGTCCACGGCCAGGCTTGCGGTATCGA
CCGAACCTCCAACCCCGACGGCGCCCTCAAGGACGCCGCTAAGTACAAGGTTGT
TTCCTACGCTAAGGTTGAGGTCTCCCCCTACATGCGAACCGTCCGAGTCATTGAC
GACTTCGGATCTGTCAAGCTGAAGGACGAGTCCGGCCACGACCGAGAGTTCCAG
GCCATTTCCGCCCAGCTGACCGCCCCCTCCATGCACACCATGGACGGCAAGCAC
TACGCCGCCGAGCTGATGGTCCTGCACAAGCCCAAGGACGACCTGGACATGCTG
CGAGAGGGCCTGATCCTGTCCGTCTTCTTCGACGACCAGAACGGCACCGAGTCC
CCCCTGTTCACCCACTTTGGATTTTCCGCCGACGGCTCCGGCAACTCCCCCTCTA
AGTCCTGGGACGCCCCCCACTACATCGACGTCGCTAAGGAGATCGAGGAGGCTG
CCAAGGGCGCCTCCTACCTCTACGACGGCTCCATGCCCGTGCCCCCCTGCCACG
AGAACATCAAGTACCTCGTGCTCGGCCACGCCGTCCCCGTCCTGCCTGCTCAGA
CCAAGGCCCTGGAGGAGACCCTGCGATGCTGGGCCGGCGGTAAGGACAAGCGA
GCCACCGTCAAGGGCACCTGCCGAACCGTCACCAAGGACTCCCTGACCCTGGGC
GGCCCCCACCACGGTGCTACCTGTAAGGCCGCCGAGGAGAAGGGTCAGTCCCA

CCGACTGGCCGCTTGCTGGGACGCCGGACTGCTGCCTGAGGAGGCCAAGTCTTG
CGTGAAGTCTCCCATCGACCTCCTGCCTACCATGGCCTCCACCACCTCCGAGGAC
CGAAAGCCCTACTTCAACTTCCGACCTGTGAAGCACGCCCACATCGCCCCCTCC
AACTTCTCTCTGGACGTCACCCCCCTGGAGATGGGCATGCCCGGTCCCCTGCCCA
ACTTTGGAACCATCATGATCCTCGGCAAGAAGTACATGGTCCGAAAGATCAGCG
TCCGACCCCTGTCCTCCCACACCTACCACGGCGAGCGACACGTTGGCGAGATTA
TCATCGAGTCCCTGGTCTTCGGCGACGAGATCAGCACCCAGGCCGCCCTCCTGTT
CGGCCGACCTGGAGGTCCCGACCACCCCGACGCTCACGGTGGACACGGCGGTG
GACACTCCGCCCACCGACGACTGAAGTACCAGCACAACAAGGACGACACCTAC
GGTGACGACGAGCTGCACCGACTGTGGATCTCCGTCCCCATTAAGCTCGGCGTC
GAGAACGCCCTGCTCCGACAGATCGGCCTGCCCTTCCAGGCCTACAAGGAGGCC
ATCAAGGACCAGCACCCCTACCAGATCGAGAACACCATCGACCTGGAGGGCGG
AATCAAGGCCGCCCTGGCTGGAAACTGGCTCTTCTACTCCGGCGGCGCCGTCGA
CGCCTCCTGTCCTAAGTGGGGCGTCCGATGGATCGCCCTGACCACCCCCATTACC
GCCTCCCTGACCCAGCTGAACTACCTGCAGCTGCCCGTCTCCGGCGTCGACTCCG
TTCGATACCCCCAGATTACCTTCACCAAGCAGGAGTACCCCCAGCAGGTCTTCA
AGAACGGCCTGCCCATCTGGGCCCTGTCCTGGCACCAGCAGTGCGACGCTAACG
CCCACTGGACCTACGACGACGTCCACTGCTGGGACGTCATCTACCCCACCTGCA
AGACCGGCACCCGACAGTCCCCTATCGACATCCTCTCCAACAAGGTCGAGACCG
TTGGCAAGGGCAACTTCCTCTCCCGAGTCGACTGGAAGCCCGTTCACGACCTGA
AGATCGCCAACAACGGCCACTCCCTGCAGGTCACCAACGACATGTTCGGCTACA
TTAAGCTGATCGGCGAGGACGGCTTCCCCGACTTTTACGACGTCGCCCAGTTCCA
CCTGCACATGCCCTCCGAGCACCTGATCGACGGCCGACAGTTCTCCGCCGAGCT
CCACGTCGTCCACACCCGACAGGTCGCCGTCGGCCAGCAGAAGAACACCTACG
ACTCCTTTCCCCTCGTCGTCCTCGGCTTTATGTTCGACATTGGTGAGGAGGAGTCC
CACTTTCTCAAGCAGTTCTACCTCGGTGAGGAGACCATCCCTAACAACACCTAC
AAGACCGCCCGACGACCCATCGACCTGATGCGATCCCTCGGCCCCGCCCTGAAC
GGCAACTTCTACCGATACGACGGCTCTTTTACCACCCCCGACTGCCACGAGGAG
ATCAAGTGGTTCGTCTTTGACCACGTCTTCTCCATGTCCCTCGCCCAGTGGGAGA
CCTTCAAGGTCGAGTTCCCCTTCGCCCACTTCAACCGACCCGTCAACCAGATTCA
CAACCACCACGTCGTCAAGAACGACTTCAAGGAGGGCGTCGAGGCCCGATACG
ACTTTTTTCTGAACCGAGACCAGGGCCGAGACCGAATGCTGCCCGGCGAGGGTT
ACATCCTGTTCCCCGTCCTCGCCTCCCTCGTCGTGATGGCCTGTATTATGCTGGCC
GTCTTCGTCCGAGAGGGCCGATCTAAGCTCGAGTCCGCCGGCGGCCTCACCGAG
ACCATCGGTCGAGTCACCTACAACCGAATGTGA

***dnCA*:**

ATGTCCTCCATCGCCCGAGGCGTCCTGGCCTCTTTTTGTCTCGTCGTCACCGC
CGCCGACAACGACGAGCGAAAGCTCCAGGCCTACGGTGGCTGCCCCATGTGGG
ACAACACCTCCTTCTCCGACGAGCCCTCCCCCTTTGGCTGGCCCCTGTCCCCTAA
CGACTGGTCCTTCGCCGAGGAGGGCGAGTGGCCCCACCACTACCTGGCCTGTGG
CGGCGTTCGACAGTCCCCCGTTAACATTCCCCTCCACGGTGACACCTGCGGCATC
AACCGAATCGGTGAGCCCGACGGAGCCCTGAAGGACGCTACCCGATACAAGGT
CGTGTCTTACGCTAAGGTTGAGGCCTCCCACTACATGCGAACCGTCAAGGTCAT
GGACGACTTCGGCACCCTGACCCTGAAGGACGAGTCCGGCAACGACGTCGTCTA
CGAGGCCATTTCCGCCCAGCTGACCGCCCCCTCCATGCACACCGTCGACGGCAA
GCACTACGCCGCCGAGCTCCTGGTCCTCCACAAGCCCATGGGTGCCCGAGACAT
GCTGCAGGAGGGAGTCATCCTGTCCGTCATGTTCGACGACACCAACGGCACCGA
GTCCCCCCTGTTCACCCACTTTGGCTTCGCCCCCGACGGCCAGGGCCACTCCCCT
TCCAAGACCTGGGCCGCCCCCCACTACATCGACGTCGCCCAGGAGATCAAGGA
GGTTGTCAAGGGCCCCTCCTACCAGTACGACGGATCTCAGCCCGTGCCCCCCTG

CCACGAGAACATCAAGTACCTGGTCCTGGGCAACGCCATCCCCGTGCTGCCCGC
TCAGGCCCTGGCTCTGGAGGAGACCCTGAAGTGCTGGGCTGGCGGCAAGCTCAA
GCGACCCCCTGTCAAGGGCGAGTGCCGAGAGATTCGAAAGGACACCACCACCC
TGGGCGGCCCCCACTTTGCCGCTACCTGTGAGGCCGCCGAGGCTGCTGGTACTTC
TTACCGACTGGCCGCCTGTTGGGACCTGGGCCTGGCTGAGGACGAGGCCGCTTC
CTGCGTCAAGTCCCCCATCGACCTGAACCAGGAGATGGCCTCCACCAAGGAGTC
TACCAAGCCCACCTTTAACTTTGCTTCCATCAAGCACGCTCGAGTGGAGCCCTCC
AACTTCTCCCTGGACGTCATCCCCCTCGAGGTCGGCGCCCCTGGTCCTCTGCCTA
ACTTCGGCACCATCATGATCCTGGGAAAGAAGTACATGGTCCGAAAGGTCTCTG
TCCGACCCCTCTCCTCCCACACCTACGAGGGCAAGCGACACGTTGGTGAGATTA
TCATCGAGGCCCTGGTTTTCGGCGACGAGATCAGCACCCAGGCCGCCATGATCT
TCGGTCAGGCCGCCAAGGAGGGCCACTCCAACGGCCACTCTAACTCCCACCGAC
GACTGAAGTACGCCCAGAACAAGGACAACACCTACGGCGACGACGAGCTCCAC
CGAGTCTTTATTTCTGTCCCCATCAAGTTCGGCGTCGAGTCCGCTCTCCTCCGACA
GATCGGCCTGCCCTTCCAGGCCTACAAGGAGGCCATCAAGGACCGACACCCCTA
CCACATCGAGTCCACCATCGACCTGCAGGGCGGCATCCAGGAGGCTCTGAACG
GCAAGTGGCTCTTCTACTCCGGCGGCGCCGTTGACCCCACCTGTCCTAAGTGGGG
AGTCCGATGGCTCGCCCTGACCACCCCCATTACCGCCTCCCTGACCCAGATTAAC
TACCTGCAGCTGCCCGTCACCGGCATGGACTCCGTTCGATTTCCCGGCATCACCC
TGAACCGACAGGAGTACGCCGAGCAGGTCTACCTCGACGGCCTGCCCATGTGGG
CCCTGTCCTGGCACAAGACCTGCGACCCCAACGCCCACTGGACCTACGACGACG
TTCACTGCTGGGACGTTATGTACCCCACCTGCAAGGAGGGCACCCGACAGTCCC
CTATCAACATCCTCACCGACCAGGTGGAGAAGGTGGGCAACTCCGACTTCCTGT
CCCGAGTGGACTGGAAGCCCGTGTCTGGCCTGAAGGTCGCCAACAACGGTCACT
CCCTGCAGATCACCAACGACATGTTCGGATACATTCGACTGACCGGTGAGGACG
GATTTCCCGACTTTTACGACATCGCCCAGTTTCACCTGCACATGCCCTCCGAGCA
CCTCATCGACGGCCGACAGTTCTCCGCCGAGCTGCACGTCGTCCACACCCGACA
GGTGGCTGTTGGCCAGGAGAAGAACACCTACGACTCCTTCCCCCTGGTTGTCCTC
GGCTTTATGTTCGACATTGGTGAGGAGGAGTCCCCCTTTCTGAAGCAGTTTTACC
TCGGTCAGGAGACCATTCCCAACAACACCTTCAAGACCACCAAGCACCCCGTCG
ACCTGATGCGATCCCTGGGCCCCGCCCTGAACGGCGACTTCTACCGATACGACG
GCTCCTTCACCACCCCCGACTGCCACGAGCAGATCAAGTGGTTCGTCTTCGACCA
CGTCTTCAAGATGTCCCTGGCCCAGTGGGAGACCTTCAAGGTCGAGTTTCCCTTT
GCCCACTTCAACCGACCCGTCAACAAGATCCAGGAGCACCACGTCGTCAAGAA
CGACTTTAAGGAGGGTGTCGAGGCTAAGTACGACTTTTTTCTGGGCCGAAACGT
GGGCCGAAACCGACTCCTGCCCGGCGAGGGTTACATCCTCTTCCCCGTCGTCGC
CTCCCTGGTCGTGATGGTCTGTATCATGCTGGCCGTCTTCGTTCGAGAGGGCCGA
TCCAAGCTGGAGTCCGCCGGCGGTCTCACCGAGACCATCGGCCGAGTCACCTAC
AACCGAGCCTAA

***cgiCA*:**

ATGAAGTACCTGCTGGCCGTCTTCGTCATCCTGGAGTCTTTCCTGTCCATCGA
GGCCGCTGCTGACAAGTGGAACTACACCGACCAGGCCTCCTGGTCTAAGATGAC
CGGTTCCCAGTGCTCCGGCACCTCTCAGTCTCCTATCAACCTGCCCCCTATGGTT
GAGATGGAGTACTCCAAGAACCTCAAGCCCTTCCAGTTCTCCGGTTACGAGGTC
GTCGTCACCGACCCTGTTGTTCACAACAACGGCCACACCATCCAGGTCGGTTTCA
CCAACGACGCCTCCATTTCCGGCGGAGACCTGGGTCAGACCTTCAAGGCTGCTC
AGTTTCACTTCCACTGGGGATCTGACAACACCAAGGGCTCTGAGCACACCTACA
ACGGCAAGTCCTACCCCGCTGAGCTGCACATTGTCCACTACAACACCAAGTACC
CCTCCCTGACCGAGGCTGTTGACAAGGTTGACGGCCTGGCTGTCCTGGGTTTTTT
CATCGAGGTCGGCCCCATCCACAACTGCAACTTTGGCCCTGTCGTCGACGCCCTG
ACCAACGTTCAGCCTGGTGTTTCCCAGTACTCCATCAACACCACCGCCAACCCCT

TTAAGCTGCGACACGTCCTGCCCATGCAGCTGACCGACTACTACCGATACATGG GCTCCCTGACCACCCCTACCTGTCTGGAGACCGTTAAGTGGACCGTCTTCCGAGA CCTCCTCTACATGTCCGAGGAGCAGCTGCAGAAGCTCCGATCTGTGTACTTCGAC TCCTCCAAGACCATGCAGATGGTCGACAACTGGCGACCCCCTCAGCCTCTGAAC GGTCGAAAGGTTTACATCTCCTTTGACGTGAAGGCCAACTCCTCCCCCACCCCTG TTTTTTCCTCCGCTCTGTTCCTGGTCCTGCTGTGTATCCACCGAGTCTTCATCTAA

***dsaCA*:**

ATGGTCTCCGAGCCCCACGACTACAACTACGAGAAGGTCGGCTTCGACTGG ACCGGCGGCGTCTGCGTCAACACCGGCACCTCCAAGCAGTCCCCCATCAACATC GAGACCGACTCCCTGGCCGAGGAGTCCGAGCGACTGGGCACCGCTGACGACAC CTCCCGACTGGCCCTGAAGGGTCTGCTGTCCTCCTCCTACCAGCTGACCTCTGAG GTCGCCATCAACCTCGAGCAGGACATGCAGTTTTCCTTTAACGCCCCCGACGAG GACCTGCCCCAGCTGACCATCGGCGGCGTCGTCCACACCTTTAAGCCCGTCCAG ATCCACTTCCACCACTTCGCCTCCGAGCACGCCATCGACGGCCAGCTGTACCCC CTGGAGGCCCACATGGTTATGGCCTCCCAGAACGACGGCTCCGACCAGCTGGCC GTCATTGGCATCATGTACAAGTACGGTGAGGAGGACCCCTTCCTGAAGCGACTC CAGGAGACCGCCCAGTCCAACGGCGAGGCCGCTGACAAGAACGTGGAGCTGAA CTCCTTCTCCATCAACGTCGCCCGAGACCTGCTGCCTGAGTCCGACCTCACCTAC TACGGCTACGACGGATCTCTGACCACCCCCGGATGCGACGAGCGAGTCAAGTGG CACGTCTTCAAGGAGGCCCGAACCGTGTCCGTCGCCCAGCTCAAGGTGTTTTCCG AGGTCACCCTGGCCGCCCACCCCGAGGCTACCGTTACCAACAACCGAGTCATTC AGCCCCTGAACGGTCGAAAGGTGTACGAGTACAAGGGCGAGCCCAACGACAAG TACAACTACGTCCAGCACGGCTTCGACTGGCGAGACAACGGCCTGGACTCCTGC GCCGGTGACGTCCAGTCCCCCATTGACATCGTGACCTCCACCCTGCAGGCCGGC TCCTCCCGATCCGACGTCTCCTCCGTCAACCTGAACGACCTGAACACCGACGCCT TCACCCTGACCGGTAACACCGTCAACATCGGCCAGGGCATGCAGATCAACTTCG GAGACCCCCCCGCCGGCGACCTGCCTGTCATTCGAATCGGCACCCGAGACGTCA CCTTCCGACCCCTGCAGGTCCACTGGCACTTCTTCCTGTCTGAGCACACCGTCGA CGGCGTCCACTACCCCCTCGAGGCTCACATCGTCATGAAGGACAACGACAACCT GGGCGACTCCGCCGGCCAGCTCGCTGTTATCGGCATCATGTACAAGTACGGAGA CGCCGACCCCTTTATTACCGACATGCAGAAGCGAGTCTCCGACAAGATCGCTTC CGGAGCCATCACCTACGGCCAGTCTGGCGTCTCTCTGAACAACCCCGACGACCC CTTCAACGTGAACATTAAGAACAACTTCCTGCCCTCCGAGCTGGGTTACGCCGG CTACGACGGCTCTCTGACCACCCCTCCCTGCTCCGAGATCGTCAAGTGGCACGTT TTCCTGGAGCCCCGAACCGTCTCCGTCGAGCAGATGGAGGTCTTTGCCGACGTG ACCCTGAACTCCAACCCCGGCGCCACCGTCACCACCAACCGAATGATCCAGCCC CTGGAGGGTCGAACCGTTTACGGCTACAACGGCGCCGCCGCCTAA

***sazCA*:**

ATGAAGAAGTTCATCCTGTCCATCCTGTCCCTGTCCATCGTCTCCATCGCCG GTGAGCACGCTATCCTCCAGAAGAACGCCGAGGTCCACCACTGGTCCTACGAGG GTGAGAACGGCCCTGAGAACTGGGCTAAGCTCAACCCCGAGTACTTTTGGTGTA ACCTGAAGAACCAGTCCCCCGTCGACATCTCCGACAACTACAAGGTCCACGCCA AGCTGGAGAAGCTGCACATCAACTACAACAAGGCCGTCAACCCCGAGATCGTC AACAACGGCCACACCATCCAGGTCAACGTCCTGGAGGACTTTAAGCTGAACATC AAGGGCAAGGAGTACCACCTGAAGCAGTTCCACTTCCACGCCCCCTCCGAGCAT ACCGTTAACGGAAAGTACTACCCCCTCGAGATGCACCTGGTCCACAAGGACAA GGACGGAAACATCGCTGTCATTGGCGTCTTCTTCAAGGAGGGCAAGGCCAACCC CGAGCTGGATAAGGTCTTCAAGAACGCCCTGAAGGAGGAGGGCAGCAAGGTCT TCGACGGCTCTATCAACATCAACGCCCTGCTGCCCCCCGTCAAGAACTACTACA CCTACAGCGGCAGCCTGACCACCCCTCCTTGTACTGAGGGCGTGCTGTGGATTGT CCTGAAGCAGCCCATCACCGCCTCGAAGCAGCAGATCGAGCTCTTTAAGTCCAT

CATGAAGCACAACAACAACCGACCCACCCAGCCCATTAACTCCCGATACATCCT
CGAGTCCAACCACCATCACCATCACCATTAA

*The underlined sequence is the secretory signal peptide.

***vgb*:**

ATGTTGGATCAACAGACCATTAACATCATCAAAGCCACTGTTCCTGTATTGA
AGGAGCATGGCGTTACCATTACCACGACTTTTTATAAAAACTTGTTTGCCAAACA
CCCTGAAGTACGTCCTTTGTTTGATATGGGTCGCCAAGAATCTTTGGAGCAGCCT
AAGGCTTTGGCGATGACGGTATTGGCGGCAGCGCAAAACATTGAAAATTTGCCA
GCTATTTTGCCTGCGGTCAAAAAAATTGCAGTCAAACATTGTCAAGCAGGCGTG
GCAGCAGCGCATTATCCGATTGTCGGTCAAGAATTGTTGGGTGCGATTAAAGAA
GTATTGGGCGATGCCGCAACCGATGACATTTTGGACGCCTGGGGCAAGGCTTAT
GGCGTGATTGCAGATGTGTTTATTCAAGTGGAAGCAGATTTGTACGCTCAAGCTG
TTGAATAG

***kar2*:**

ATGAAGTTCTCTATGCCTTCGTGGGGCGTCGTTTTTACGCCCTCCTGGTATG
CCTTCTGCCTTTCCTTTCCAAGGCCGGCGTTCAGGCTGATGACGTGGACTCTTATG
GCACCGTCATTGGTATCGATCTGGGTACCACCTACTCCTGTGTTGGTGTCATGAA
GGGTGGCCGAGTCGAGATTCTGGCCAACGACCAGGGTTCTCGAATCACCCCCTC
CTACGTGGCCTTCACCGAGGACGAGCGACTCGTTGGAGACGCTGCCAAGAACCA
GGCTGCCAACAACCCTTTCAACACCATTTTCGACATTAAGCGACTCATTGGTCTT
AAGTACAAGGACGAGTCCGTCCAGCGAGACATCAAGCACTTCCCCTACAAGGT
CAAGAACAAGGACGGCAAGCCCGTTGTTGTCGAGACCAAGGGCGAGAAGA
AGACCTACACCCCCGAGGAGATCTCCGCCATGATTCTTACCAAGATGAAGGACA
TTGCCCAGGACTACCTTGGCAAGAAGGTCACCCACGCTGTCGTCACCGTCCCTG
CCTACTTCAACGATGCCCAGCGACAGGCCACCAAGGATGCCGGTATCATTGCTG
GTCTCAACGTTCTGCGAATTGTTAACGAGCCCACCGCTGCCGCCATTGCCTACGG
CCTGGACCACACCGATGACGAGAAGCAGATTGTTGTCTACGATCTTGGTGGAGG
AACCTTCGATGTTTCTCTTCTGTCTATCGAGTCTGGTGTCTTTGAGGTTCTTGCCAC
TGCTGGTGACACCCATCTTGGTGGTGAGGATTTCGACTACCGAGTCATCAAGCAC
TTTGTCAAGCAGTACAACAAGAAGCACGACGTCGACATTACCAAGAACGCTAA
GACCATTGGTAAGCTCAAACGAGAGGTTGAGAAGGCCAAGCGAACTCTGTCTTC
CCAGATGTCCACTCGAATCGAGATTGAGTCCTTCTTCGATGGAGAGGACTTCTCG
GAGACCCTGACCCGAGCCAAGTTCGAGGAGCTCAACATTGATCTGTTCAAGCGA
ACCCTCAAGCCCGTTGAGCAGGTTCTCAAGGACTCTGGCGTCAAGAAGGAGGAT
GTCCACGACATTGTTCTTGTTGGTGGTTCCACCCGAATCCCCAAGGTCCAGGAGC
TGCTGGAGAAGTTCTTTGACGGCAAGAAGGCCTCCAAGGGTATCAACCCCGATG
AGGCTGTTGCTTACGGAGCTGCTGTCCAGGCTGGTGTTCTTTCCGGCGAGGACGG
TGTTGAGGACATTGTCCTGCTCGATGTTAACCCCCTGACTCTTGGTATTGAGACC
ACTGGTGGTGTCATGACCAAGCTCATCAACCGAAACACCAACATCCCCACCAAG
AAGTCCCAGATTTTCTCCACCGCTGTTGACAACCAGTCTACCGTGCTGATTCAGG
TCTTTGAGGGAGAGCGAACCATGTCCAAGGACAACAACCTGCTTGGTAAGTTCG
AGCTCAAGGGTATTCCCCCTGCTCCCCGAGGTGTCCCCCAGATTGAGGTCACCTT
CGAGCTTGACGCTAACGGAATTCTGCGAGTCACCGCCCACGATAAGGGCACCGG
CAAGTCCGAGACCATTACCATCACCAACGACAAGGGCCGTCTCTCCAAGGACG
AGATTGAGCGAATGGTTGAGGAGGCTGAGCGATTTGCCGAGGAGGATGCTCTCA
TCCGAGAGACCATTGAGGCTAAGAACTCTCTCGAGAACTACGCCCACTCTCTCC
GAAACCAGGTTGCTGACAAGTCTGGTCTCGGTGGCAAGATTTCTGCCGACGACA
AGGAGGCTCTCAACGACGCTGTCACCGAGACTCTCGAGTGGCTGGAGGCCAACT
CCGTGTCTGCCACCAAGGAGGACTTTGAGGAGAAGAAGGAGGCTCTGTCTGCCA
TTGCCTACCCCATCACCTCCAAGATCTACGAGGGTGGAGAAGGTGGAGACGAGT

CCAACGACGGTGGATTCTACGCTGATGATGATGAGGCTCCTTTCCACGATGAGCT TTAA

pdi:

ATGAAGTTCACTGCCCTCACAATTGCGCTCATGGGCGCTCTGGCTGCGGCCT CCGACGTCGTCAAGCTCGATTCCGACAACTTTGCCGACTTTGTCACTGACAACAA GCTCGTCCTCGCTGAGTTCTTTGCTCCCTGGTGCGGACACTGCAAGCAACTCGCC CCCGAGTACGAGTCTGCAGCCACCATCCTTAAGGAGAAGGGCATCCCCATTGGA AAGGTCGACTGTACCGAGAACGAGGAGCTCTGCTCCAAGTTTGAGATCCAGGGC TACCCCACCCTCAAGATCTTCCGTGGATCTGAGGAGGACAGCTCTCTCTACCAGT CTGCCCGAACCTCCGAGGCAATTGTCCAGTACTTGCTGAAGCAGGCCCTTCCTCT CGTTTCCGAATTCGCCAACGAGAAAGAGCTCAATGCCTTCACCAAGGACAACGA CGTCACCATTGTTGCCTTCCACGATGAGGATGACGAGAAGTCTCAGTCCACCTTC CAGCGAGTCGCACAGAAGCTCCGAGAGCGATTCACTTTCGGCCACTCCGCTGAC AAGGCCCTTGCCAAGAAGTACGGTGTCGAGAAGTTCCCCGCTCTCGTTGTCTACC GAAACTTCGATGAGAAGCCCGCCGTTTACGACATCTCTGCTGGCAAGAAGGTGT TCAAGTTTAAGCCCGAGCCTCTTACCAAGTTCATCAAGACCGAGGCTGTCCCCGT CATTGGTGAGATTGGTCCCGCTTCTTTCCAGGACTACGCCACATCTGGCCTCCCT CTGGTCTACATCTTCTCTGCTCTGGAGAAGGACACCAAGCAGATCTCCGAGTGG GTCAAGCCTTGGGCTGAGAAGCTGAAGGGTGAGGCTTATGTCGGTGTCATTGAC GCTGACCTCTACGGATCTCACGCCCAGAACGTCAACATCCAGGAGAAGTTCCCT GCCATCGCTATCGAGAACTTCGACAACAAGAAGAAGTGGGCTCATGCTCAGGAT GCCAAGATCACCAAGGCCTCCGTCGACAAGTTCTTCAAGGAGTACATTGAGGGA ACTCTCGAGCCTATCCTCAAGTCTGACCCCGTCCCCGAGTACCAGGATGGTCCCG TCCACATTGTTGTCGGCAAGAACTACAAGGATATTGTTCTCGATGATGACAAGG ATGTTCTGATCGAGTTCTACGCTCCCTGGTGCGGACACTGCAAGATTCTTGCTCC CATCTACGACGAGCTTGGAGACCTCTTCTTCGACCACCCCGAGATCTCCAAGAA GGTTACTGTCGCCAAGATCGACGCCACCACTAACGAGTTCCCTGATGAGGATGT CAAGGGTTTCCCCACTATCAAGCTGTACCCCGCCGGTAAGAAGAACGCCCCTAT CACCTACCCCGGTGCTCGAACCCTTGAGGGTCTTAACCAATTCATCAAGGAGCA CGGTACCCACAAGGTTGACGGTCTCGCCCATGCCGATGAGGAGGAGGCCCCTGC CAAGGATACCAAGGCCAAGAAGGGAGGCAAGATTGATGATGAGCTTTAA

rpl3:

ATGTCCCACCGAAAGTACGAACAGCCCCGACACGGTCACCTGGGTTTCCTC CCCCGAAAGCGATGCACCAAGTCTCGAGGAAAGGCCAAGTCTTTCCCCAAGGA TGACAAGTCCAAGCCTGTTGCTCTGACCGCCTTCCTTGGTTACAAGGCCGGTATG ACCACCATTGTCCGAGACCTCGACCGACGAGGCTCCAAGATGGACAAGCGAGA GATTGTCGAGGCTGCCACCGTCGTTGACACTCCCCCCATGGTTGTTGTCGGTGTT GTTGGTTACGTTGCCACTCCCCGAGGTCTTCGATCCCTGACCACCGTCTGGGCCG AGCACCTGTCCGAGGAGGTCCGACGACGATTCTACAAGAACTGGTACAAGTCCA AGAAGACTGCCTTCACCAAGTACGCTAAGCAGTACGAGGGTGACGCCGCCCAG GTCCAGAAGGAGCTCGAGCGAATCAAGAAGTACTGCACCGTTGTGCGAGTTCTT GCTCACACCCAGCCCAAGCTGACTCCTCTTAAGCAGAAGAAGGCTCACCTTGCT GAGATCCAGATCAACGGTGGCTCCGTTGCCGACAAGGTCGAGTGGGCCCAGCA GCACTTTGAGAAGACCGTCACCATCGACACTGTTTTCGAGCAGGACGAGATGAT TGACGCCATTGCCATCACCAAGGGTAAGGGATACGAGGGTACCACCCACCGAT GGGGAACCAAGAAGCTGCCCCGAAAGACCCACCGAGGTCTGCGAAAGGTCGCC TGTATTGGTGCCTGGCACCCTGCCAACGTCCAGTACACCGTGGCTCGATCCGGTC AGGACGGTTACCACCACCGAACCTCCGCCAACCACAAGGTTTACCGAGTTGGCA AGGGCGGTGACGAGTCCAACGGCTCCACCGAGTTCGACCGAACCCCCAAGACC

ATCACCCCCATGGGTGGTTTCGTCCGATACGGTGAGGTCAACAACGACTTCCTCA
TCCTCAAGGGTTCCATCCCCGGTGTCAAGAAGCGAGTCATCACTCTGCGAAAGT
CCATGTGGCTGCACACCTCTCGACGAGACCTCGAGAAGACCCAGCTCAAGTGGA
TCGACACTGCCTCCAAGTTCGGAAAGGGCCGATTCCAGACCCCTGCCGAGAAGA
CTGCTTTCATGGGCACTCTCAAGAAGGACCTTTAA

lip2* from *Y. lipolytica:

ATGAAGCTTTCCACCATCCTCTTCACAGCCTGCGCTACCCTGGCTGCCGCCC
TCCCTTCCCCCATCACTCCTTCTGAGGCCGCAGTTCTCCAGAAGCGAGTGTACAC
CTCTACCGAGACCTCTCACATTGACCAGGAGTCCTACAACTTCTTTGAGAAGTAC
GCCCGACTCGCAAACATTGGATATTGTGTTGGTCCCGGCACTAAGATCTTCAAGC
CCTTCAACTGTGGCCTGCAATGTGCCCACTTCCCCAACGTTGAGCTCATCGAGGA
GTTCCACGACCCCCGTCTCATCTTTGATGTTTCTGGTTACCTCGCTGTTGATCATG
CCTCCAAGCAGATCTACCTTGTTATTCGAGGAACCCACTCTCTGGAGGACGTCAT
AACCGACATCCGAATCATGCAGGCTCCTCTGACGAACTTTGATCTTGCTGCTAAC
ATCTCTTCTACTGCTACTTGTGATGACTGTCTTGTCCACAATGGCTTCATCCAGTC
CTACAACAACACCTACAATCAGATCGGCCCCAAGCTCGACTCTGTGATTGAGCA
GTATCCCGACTACCAGATTGCTGTCACCGGTCACTCTCTCGGAGGAGCTGCAGCC
CTTCTGTTCGGAATCAACCTCAAGGTTAACGGCCACGATCCCCTCGTTGTTACTC
TTGGTCAGCCCATTGTCGGTAACGCTGGCTTTGCTAACTGGGTCGATAAACTCTT
CTTTGGCCAGGAGAACCCCGATGTCTCCAAGGTGTCCAAAGACCGAAAGCTCTA
CCGAATCACCCACCGAGGAGATATCGTCCCTCAAGTGCCCTTCTGGGACGGTTA
CCAGCACTGCTCTGGTGAGGTCTTTATTGACTGGCCCCTGATCCACCCTCCTCTCT
CCAACGTTGTCATGTGCCAGGGCCAGAGCAATAAACAGTGCTCTGCCGGTAACA
CTCTGCTCCAGCAGGTCAATGTGATTGGAAACCATCTGCAGTACTTCGTCACCGA
GGGTGTCTGTGGTATCTAA

*The underlined sequence is the secretory signal peptide.
